# Genome-wide DNA methylation profiling of triple-negative breast cancer uncovers epigenetic biomarkers of tumor identity and the immune microenvironment

**DOI:** 10.1038/s41523-026-01030-y

**Published:** 2026-08-12

**Authors:** Iñaki Sasiain, Deborah F. Nacer, Mats Jönsson, Johan Vallon-Christersson, Anita Grigoriadis, Mattias Aine, Johan Staaf

**Affiliations:** 1 Division of Translational Cancer Research, Department of Laboratory Medicine, Lund University, Lund, Sweden; 2 Lund University Cancer Center, Lund University, Lund, Sweden; 3 Division of Oncology, Department of Clinical Sciences Lund, Lund University, Lund, Sweden; 4 Cancer Bioinformatics, Breast Cancer Now Unit, Comprehensive Cancer Centre, Faculty of Life Sciences and Medicine, King’s College London, London, UK; 5 Spatial Biology Facility, Faculty of Life Sciences and Medicine, King’s College London, London, UK

**Keywords:** Biomarkers, Cancer, Computational biology and bioinformatics, Genetics, Oncology

## Abstract

DNA methylation deregulation is an essential feature of tumor biology, influencing cancer formation and pathogenesis. Analyses of DNA methylation in bulk cancer specimens are challenging due to the high data dimensionality, noise, and mixture of different cell types. Here, we characterized methylation dynamics in cancer by investigating the variance structure of bulk tumor DNA methylation data through the identification of groups of highly correlated CpGs, termed CpG cassettes. Using triple-negative breast cancer as a model system, our approach identified co-occurring methylation patterns linked to different intrinsic tumor processes and pathways, gene inactivation, and composition of the tumor immune microenvironment. Our framework also demonstrated the presence of high-variance DNA methylation, seemingly not linked to tumor biology, that could be excluded using chromatin accessibility filtering. Together, this work outlines a comprehensive approach to analyze bulk tumor DNA methylation data, combining tumor purity adjustment, functional CpG filtering, stratification by CpG contexts, and identification of highly correlated CpG modules to enhance tumor-intrinsic DNA methylation patterns and our understanding of processes shaping epigenetic tumor evolution.

## Introduction

Epigenetic mechanisms, such as DNA methylation and histone acetylation, regulate gene expression without modifying the genome. Deregulation of these processes is acknowledged as a major driver of cancer formation and pathogenesis^1^. DNA methylation involves the covalent addition of methyl groups in cytosine residues located at cytosine-guanine dinucleotides (CpG) across the genome and plays a vital role in transcriptional regulation, gene imprinting, X-chromosome inactivation, and suppression of repeat elements^2^. In the context of cancer, DNA methylation has been involved in several relevant molecular mechanisms, like genome repair^3,4^, immune evasion^5,6^, and treatment response^7,8^. A deeper understanding of DNA methylation dynamics in cancer and how it shapes the tumor ecosystem is, therefore, a highly relevant aspect of cancer research.

DNA methylation profiling of bulk tumor tissue is frequently performed by high-density microarrays due to their cost-effectiveness, ease of use, and high CpG coverage^9^. However, these high-dimensional data constitute a complex data modality influenced by both intrinsic confounders, such as analytical noise and tumor heterogeneity, and extrinsic factors like the presence of non-malignant cells with cell-type specific methylation signatures. Together, these factors cause variation in the methylation estimates of single CpGs (referred to as beta values), where tumor heterogeneity and tumor purity have been highlighted as important sources of bias in analyses if not accounted for^10^. Given the complexity of DNA methylation data, different analytic frameworks have been implemented in the context of cancer. A common strategy is variance-based filtering, which reduces dimensionality and noise by retaining only the *N* most variable CpG sites before unsupervised or supervised analyses^11–15^. This approach seeks to remove non-informative CpGs and enrich for those whose variability more likely reflects underlying biological processes. Variance filtering, however, also has potential drawbacks as it could keep irrelevant high-variance CpGs and is influenced by tumor extrinsic confounders, like the normal cell fraction. To circumvent the latter, tumor purity adjustment methods have been developed that can be applied before variance-based filtering to enrich for tumor-intrinsic signals^16–18^.

An additional layer of biological complexity in the analysis of DNA methylation data arises from CpG contexts, i.e., the genomic context in which the CpGs are located. CpGs can be divided into three broad contexts based on their relative location to genes: (i) promoter CpGs, affecting the promoter regions of genes, (ii) proximal CpGs, located close to the promoter regions but not in the core promoter sequence, and (iii) distal CpGs, located at a greater distance from gene promoters. CpG context has functional implications; while promoter and proximal CpGs are often involved in the regulation of transcription factor binding to gene promoters, directly affecting gene expression, distal CpGs have a less straightforward link to genes and can be involved in regulating the binding of transcription factors to enhancer regions^2,19,20^. In addition, CpGs can reside in regions with different chromatin accessibility, normally referred to as open (accessible) or closed (inaccessible) chromatin as measured by, e.g., the Assay for Transposase-Accessible Chromatin using sequencing (ATAC-seq) method.

Given the complexity of bulk tumor DNA methylation data, this study aimed to deconstruct and characterize its variance structure, considering both the genomic context of CpGs and the adjustment of methylation data for tumor purity. Our hypothesis is that this approach can identify groups of CpGs (referred to as CpG cassettes) that share common methylation patterns across samples and are likely involved in related or co-occurring biological processes. As a model system for these analyses, we focused on triple-negative breast cancer (TNBC) based on recent work that demonstrated the importance of both tumor purity correction and CpG context for unsupervised analysis of DNA methylation data^21^. TNBC constitutes ~10–20% of breast cancer cases and is characterized by the negativity of three molecular markers: the HER2/ERBB2 oncogene and the progesterone (PR) and estrogen receptors (ER)^22^. This subtype is highly heterogeneous, with an often aggressive clinical course, shows high genomic instability, and has historically lacked available targeted therapies^23^. TNBC has been molecularly stratified by, e.g., mRNA profiling, including both the PAM50 subtypes^24^ (~70% belong to the Basal PAM50 subtype) or TNBC-specific subtypes like the Lehmann et al. subtyping scheme, dividing TNBC into Luminal Androgen Receptor (LAR), Mesenchymal (M), Basal-Like 1 (BL1), and Basal-Like 2 (BL2) tumors^25,26^. In agreement with PAM50, previous studies have demonstrated that the Basal/non-Basal division is highly relevant in TNBC DNA methylation dynamics, with clearly differentiated methylation patterns between tumors, representing the two main attractor states within the TNBC methylation landscape^21^. Besides global DNA methylation patterns, gene-specific methylation alterations also play an important role in TNBC. *BRCA1* promoter hypermethylation has been reported as the most frequent cause of a genetic homologous recombination deficiency (HRD) phenotype alongside mutations in the *BRCA1* or *BRCA2* genes^27^, and *CDH1* hypermethylation has been associated with tumor invasiveness^28^. Finally, immune infiltration introduces an additional dimension of heterogeneity in TNBC, lowering tumor cell purity in bulk specimens, while being a clinically relevant biomarker for better outcomes and response to treatment^29^. Immune infiltration in TNBC has so far been associated with features like low clonal heterogeneity, mutation load, copy number alterations^30^, and even epigenetic subtypes^21^. Despite intense research, we are still far from a mechanistic understanding of the factors driving heterogeneity in the tumor immune microenvironment (TIME), both across cancers in general and in TNBC specifically, and what role somatic DNA methylation alterations plays for the TIME composition. With the increasing usage of immune checkpoint therapy also for early-stage TNBC patients, understanding the TIME and how it is shaped becomes increasingly important. In this setting, TNBC provides an intriguing model system for deconstructing bulk tumor DNA methylation data in order to identify novel epigenetic features associated with both tumor-intrinsic and -extrinsic factors.

In this work, we demonstrate how statistical deconstruction of bulk tumor DNA methylation data can identify biologically relevant CpG sets that mirror gene-specific alterations and patterns specific to molecular subtypes, as well as variance (noise) without apparent biological relevance for the disease in question that may obscure data analyses. Moreover, by integrating additional -omics layers combined with in situ methods like spatial transcriptomics, we identify putative epigenetically driven tumor alterations that correlate with an altered TIME, providing support for a concept of tumor-intrinsic epigenetic immune evasion.

## Results

### CpG cassettes in tumor purity-adjusted TNBC DNA methylation data

**Fig. 1:**
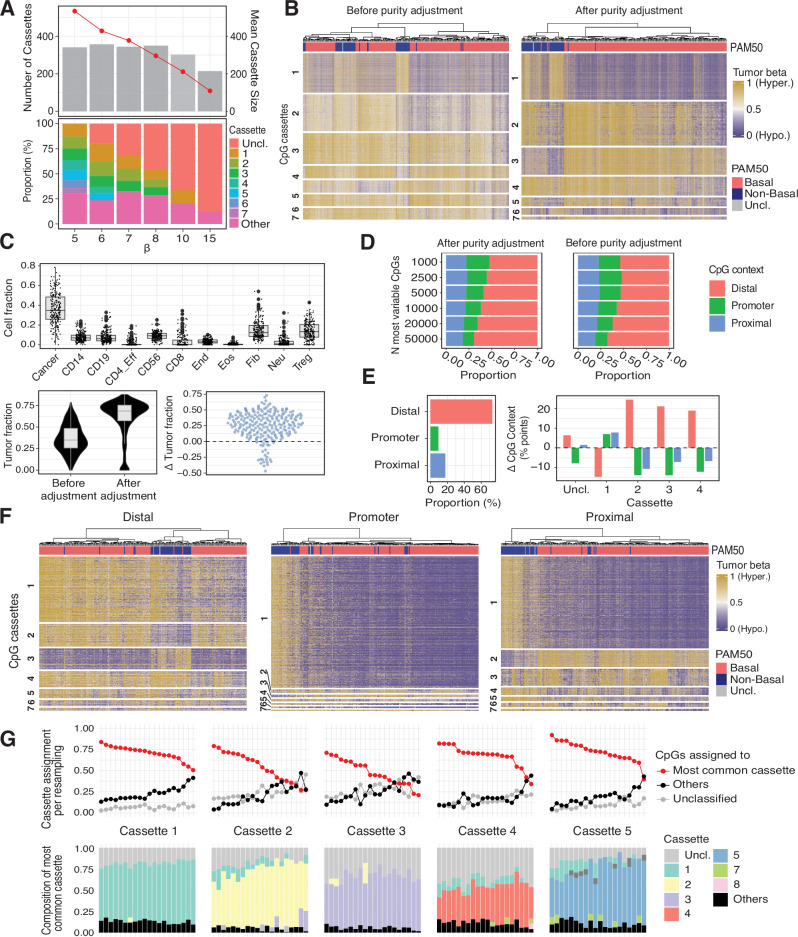
Detection of CpG cassettes in the discovery cohort. **A** Impact of the soft-thresholding parameter (*β*) on the number of detected cassettes (bars), cassette size (red line), and proportion of clustered and unclassified (Uncl.) CpGs from the 741,145 CpGs of the discovery cohort. A higher *β* results in smaller cassettes, comprising a lower portion of all CpGs, and a higher proportion of unclassified CpGs. **B** Heatmap of the first seven cassettes detected using tumor purity-adjusted and unadjusted methylation values, respectively. Columns correspond to samples and rows to CpGs. **C** Cell type admixture estimated from unadjusted beta values using MethylCIBERSORT (top) and change in tumor fraction before and after tumor purity adjustment (bottom). *CD14* stands for macrophages and monocytes, *CD19* for B cells, *CD4_Eff* for Helper T lymphocytes, *CD56* for natural killer (NK) cells, *CD8* for cytotoxic T lymphocytes, *End* for endothelial cells, *Eos* for Eosinophils, *Fib* for Fibroblasts, *Neu* for neutrophiles and *Treg* for Regulatory T-cells. **D** Proportion of CpG contexts after selecting the *N* most varying CpGs after (left) or before (right) tumor purity adjustment. **E** Distribution of CpG contexts in the input data used for cassette detection (left), and deviations in CpG context composition of the four largest detected cassettes and unclassified CpGs compared to the original distribution (right). **F** DNA methylation heatmaps for the seven largest CpG cassettes determined in distal, promoter, and proximal CpG contexts, respectively, from purity-adjusted methylation data. **G** Stability analysis of five distal cassettes detected based on 20 random samplings of 100 tumors each. Top row: for each CpG site originally assigned to a given cassette, the proportion of resamplings in which it was assigned to the most common cassette (red), to any cassette other than the most common (black), or was not assigned to any cassette (gray) is shown. A high red line indicates consistent reassignment to the same cassette across resamplings. Bottom row: proportional cassette assignments for each CpG site across resamplings, with colors representing the cassettes to which CpGs were assigned. Uniform colors within a cassette indicate strong preservation of the original group structure.

To detect CpG cassettes (groups of covarying CpGs) in TNBC, we applied Weighted Gene Correlation Network Analysis (WGCNA) on DNA methylation *M* values (representing a logit transformation of beta values) from 741145 CpGs in the 235-sample Swedish SCAN-B discovery cohort, both before and after adjusting methylation values for tumor purity, and without initially stratifying into CpG context (see Methods for details and Table 1 for patient characteristics). WGCNA clusters CpGs based on correlation values weighted with a soft-thresholding parameter (*β*) controlling the stringency of the clustering. Higher *β* values led to more homogeneous cassettes, i.e., smaller and more tightly co-methylated CpG sets that comprised a smaller fraction of all CpGs available (Fig. 1A). We selected cassettes defined by *β* = 7 for further analyses to balance cassette homogeneity and the fraction of clustered CpGs, as well as because this value produced a network with approximate scale-free topology (Supplementary Fig. 1). In a scale-free network, most nodes (CpGs) have few connections, while a few nodes serve as highly connected hubs, a property commonly observed in biological networks and considered desirable for WGCNA. To assess the impact of tumor purity adjustment on the CpG cassette detection, we compared WGCNA results for adjusted versus unadjusted methylation data. WGCNA on adjusted CpG data identified more distinct inter-cassette patterns and greater consistency across samples compared to unadjusted data (Fig. 1B). This result is presumably due to the removal of the effect of the cellular admixture from the tumor sample, as purity-adjusted data showed, as expected, higher tumor fractions based on MethylCIBERSORT analysis (Fig. 1C). Given our focus on tumor-intrinsic patterns, tumor purity-adjusted methylation data were used for all subsequent analyses.

**Table 1:** Composition and characteristics of the discovery and validation cohorts

	Discovery	Validation
***N***	235	136
**Age**	62 (IQR: 51–72)	62.5 (IQR: 50–75)
**TILs**	20 (IQR: 10-40)	ND
**Grade**		
2	26 (11.1%)	24 (17.6%)
3	206 (87.7%)	81 (59.6%)
Not available	3 (1.3%)	31 (22.8%)
**Lymph node status**		
N0	152 (64.7%)	87 (64%)
N+	83 (35.3%)	39 (28.7%)
Not available	0 (0%)	10 (7.4%)
**PAM50**		
Basal-like	187 (79.6%)	95 (69.9%)
Normal-like	7 (3%)	13 (9.6%)
HER2-enriched	34 (14.5%)	27 (19.9%)
Luminal A	5 (2.1%)	10 (7.4%)
Luminal B	1 (0.4%)	0 (0%)
Unclassified	1 (0.4%)	0 (0%)
**Lehmann TNBCtype**		
LAR	40 (17%)	32 (23.5%)
M	54 (23%)	29 (21.3%)
BL1	91 (38.7%)	29 (21.3%)
BL2	47 (20%)	20 (14.7%)
Unspecified	0 (0%)	20 (14.7%)
Not available	3 (1.3%)	6 (4.4%)
**HRD status**		
HRD	139 (59.1%)	ND
HRP	96 (40.9%)	ND
**Epitype**		
Basal1	53 (22.6%)	23 (16.9%)
Basal2	68 (28.9%)	37 (27.2%)
Basal3	54 (23%)	29 (21.3%)
NonBasal1	29 (12.3%)	22 (16.2%)
NonBasal2	31 (13.2%)	25 (18.4%)

ND stands for unavailable data.

Age and TILs are reported as median value and interquartile range (IQR) per cohort.

We next focused on the impact of CpG context (promoter, proximal, and distal) on cassette detection in the discovery cohort. CpG context is not inherently captured by variation in methylation levels across samples, nor can it be addressed by analyzing only the *N* most variable CpGs or by correcting for tumor purity (Fig. 1D). However, it plays a substantial role in methylation dynamics given its functional role in gene regulation^19,31^. Ignoring CpG context leads to the identification of CpG cassettes with substantial differences in CpG context proportions (Fig. 1E). For instance, the detected cassette 1 is enriched in promoter and proximal CpGs and shows a lower fraction of distal CpGs than the general population, while cassettes 2, 3, and 4 show the opposite pattern. This indicates that some of the detected methylation patterns are CpG context-specific. Consistently, performing WGCNA-based cassette detection within each CpG context, respectively, identified cassettes with clearly different methylation patterns (Fig. 1F). An important aspect of our CpG cassette detection approach is reproducibility and robustness. To evaluate the stability of the identified CpG context-specific cassettes in a smaller dataset, we randomly subsampled the discovery cohort data down to 100 samples (while maintaining the proposed DNA methylation epitype distribution^21^) and performed a new CpG context-aware cassette detection. This was performed 20 times. Comparison of results to the original CpG context cassettes showed that CpGs from the original distal cassettes remained largely grouped together in subsampling cassettes with similar methylation patterns as the original ones (Fig. 1G), and a similar observation was also made for proximal and promoter CpGs (Supplementary Fig. 2). Finally, the DNA methylation heatmap patterns observed for the CpG context-specific cassettes in the discovery cohort appeared similar when compared to the 136-sample SCAN-B validation cohort (Supplementary Fig. 3), and all detected CpGs cassettes except for distal cassette 106 showed moderate to high preservation in the validation cohort, as evaluated by the Zsummary statistic (Supplementary Fig. 4)^32^. Together, these results support the robustness and reproducibility of the cassette approach. The identified CpG cassettes for all, promoter, proximal, and distal CpGs obtained from adjusted methylation values using *β* = 7 are available as Supplementary Data File 1–4, respectively.

### CpG cassettes reflect major biological classes in TNBC

**Fig. 2:**
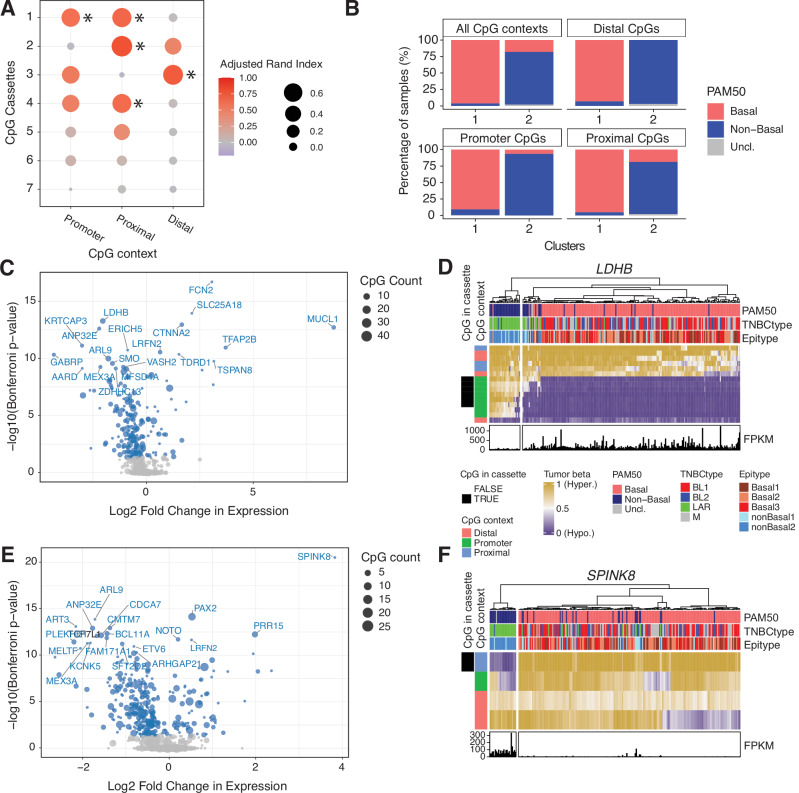
The identified cassettes reflect the Basal/non-Basal division in the discovery cohort. **A** Adjusted Rand Index (ARI) between hierarchical clustering from each of the largest CpG cassettes (Euclidean distance, Ward.D2 linkage) and a PAM50 Basal/non-Basal division. Cassettes with ARI > 0.55 are marked with asterisks. **B** Distribution of PAM50 subtypes in sample clusters obtained from beta values of all CpGs included in cassettes with ARI > 0.55 (*n* = 19770 CpGs) using a hierarchical approach (Euclidean distance, Ward.D2 linkage). **C** Volcano plot of genes with expression patterns driven by CpGs in promoter cassette 1. Differential gene expression *p* values were obtained from Wilcoxon’s tests between two clusters of samples obtained through hierarchical clustering (Euclidean distance, Ward.D2 linkage) of DNA methylation beta values of CpGs in promoter cassette 1. In the differential gene expression analysis, only genes linked to CpGs included in promoter cassette 1 were tested. The fold change in expression between sample groups was calculated using the median between both groups. **D** CpGs (rows) affecting *LDHB* and its FPKM gene expression per sample (columns). Samples were clustered based on promoter and proximal methylation patterns from CpGs linked to each gene, respectively, using k-means clustering and *k* = 2. **E** Volcano plot of genes with expression patterns driven by CpGs in proximal cassette 1. The analysis was performed as in (**C**), using clustering of CpG beta values linked to proximal cassette 1 to define the two sample groups for which differential gene expression of genes linked to the CpGs in the cassette was tested. **F** CpGs (rows) affecting *SPINK8* and its FPKM gene expression per sample (columns). Samples were clustered based on promoter and proximal methylation patterns from CpGs linked to each gene, respectively, using k-means clustering and *k* = 2. The row annotations of each heatmap indicate the CpGs included in promoter cassette 1 and proximal cassette 1, respectively.

A distinct feature of the clustered cassette heatmaps in Fig. 1 is the aggregation of tumors into mainly a PAM50 Basal and a non-Basal branch (e.g., Fig. 1F). To examine this observation in more detail, we analyzed the association of detected CpG cassettes in the three CpG contexts with major biological divisions in TNBC, in particular the Basal/non-Basal division proposed to represent the two main global methylation states in TNBC^21^. Among the seven main cassettes identified within each CpG context, several, but not all, captured the two primary methylation states in TNBC. This was quantified using the Adjusted Rand Index (ARI) between sample clusters derived from each cassette and the PAM50 Basal/non-Basal classification (Fig. 2A). Specifically, five cassettes (promoter cassette 1, *n* = 5616 CpGs; proximal cassette 1, *n* = 5322 CpGs; proximal cassette 2, *n* = 989 CpGs; proximal cassette 4, *n* = 445 CpGs; and distal cassette 3, *n* = 7398 CpGs) exhibited ARI values greater than 0.55, indicating concordance with the Basal/non-Basal split. Additionally, other cassettes, such as promoter cassettes 3 and 4, proximal cassette 5, and distal cassette 2, showed weaker but still clear capacity to separate Basal from non-Basal samples (ARI > 0.45). Hierarchical clustering of the CpGs included in high-ARI cassettes successfully differentiated Basal from non-Basal tumors (Fig. 2B).

Moreover, CpGs included in the detected cassettes were linked to biologically relevant processes based on pathway enrichment analysis, which was restricted to promoter and proximal cassettes due to available gene mappings. The largest promoter CpG cassette linked to the Basal/non-Basal division, promoter cassette 1, showed enrichment for 78 Gene Ontology terms, predominantly related to developmental processes (Supplementary Table 2). To analyze gene expression differences for genes linked to the CpGs in promoter cassette 1, we first divided samples into two groups based on hierarchical clustering of DNA methylation values of CpGs included in this cassette (conceptually representing a division into a hypo- and hypermethylated group), followed by differential gene expression analysis between the groups that was restricted to the genes linked to the included cassette CpGs. This analysis identified changes in gene expression for genes such as *LDHB*, *MUCL1*, *FCN2*, *SLC25A18*, *ANP32E*, and *TFAP2B* (Fig. 2C). As a more detailed example, *LDHB* was selected to show the expression changes linked to promoter cassette 1 (Fig. 2D). *LDHB* shows low mRNA expression in non-Basal TNBC, consistent with the hypermethylation of involved promoter CpGs, with an opposite pattern in Basal tumors. Similar findings were observed when analyzing the main proximal cassette linked to the Basal/non-Basal division, proximal cassette 1. This cassette showed enrichment of 185 GO terms, mostly including developmental and neurodevelopmental pathways (Supplementary Table 3) when the specific genes linked to the included cassette CpGs were analyzed. This cassette was also associated with expression changes in genes such as *SPINK8*, *PRR15*, *ARL9*, and *PAX2* when a similar differential gene expression analysis was performed as for promoter cassette 1 (Fig. 2E). *SPINK8* was further analyzed, showing that expression is notably correlated with the DNA methylation status of a proximal CpG, with mRNA expression only in non-Basal tumors (Fig. 2F).

### Chromatin accessibility distinguishes biologically relevant variation in distal CpGs

**Fig. 3:**
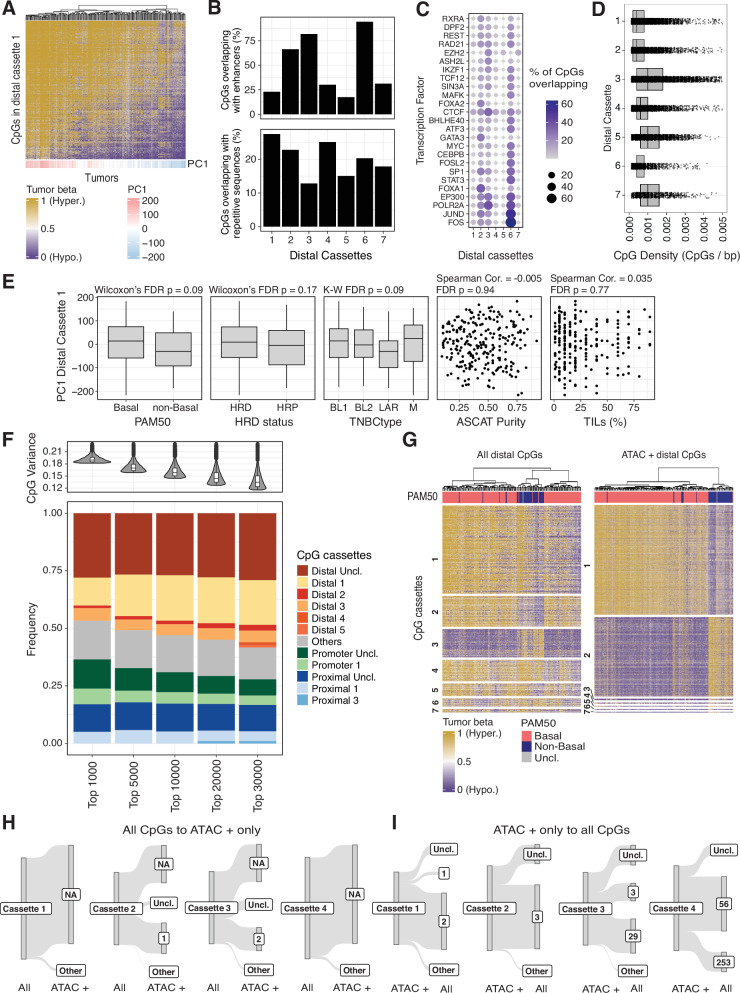
Characteristics of distal cassettes. **A** DNA methylation values of CpGs belonging to distal cassette 1 (rows) and its summary PC1 value per sample (columns). **B** Fraction of CpGs overlapping with enhancers (top panel) and repetitive regions (lower panel) in the seven largest distal cassettes. **C** Fraction of CpGs overlapping with transcription factor (TF) binding sites in the seven largest distal cassettes. TFs shown are the 25 with the highest pairwise differences between any two of the analyzed cassettes. **D** CpG density in the seven largest distal cassettes. **E** Comparison between PC1 of distal cassette 1 and several biological features. From left to right: PAM50 Basal/non-Basal, HRD, Lehmann TNBC subtypes (TNBCtype), ASCAT tumor purity estimated from whole genome sequencing, and TILs. Cor. = correlation. *P* values were corrected using the Benjamini–Hochberg (or False Discovery Rate, FDR) approach. **F** Proportion of CpG cassettes per *N* top variable CpGs after variance-based filtering on tumor purity-adjusted methylation data. The top violin plot shows the measured variance from adjusted beta values per group, and the bar plot shows the proportion of cassettes. CpGs in cassettes comprising less than 1% of the total CpGs were grouped and identified as “Others.” Uncl. = unclassified CpGs. **G** Distal cassettes detected before and after chromatin accessibility-based filtering (considering overlap with ATAC-seq peaks). **H** Sankey plots between cassettes determined from all CpGs (All) and only CpGs with ATAC-seq peak overlap (ATAC+). The NA category represents CpGs that were filtered out from the data, “Other” includes cassettes with a proportion below 2%, and “Uncl.” includes unclassified CpGs. **I** Sankey plots between cassettes determined from ATAC-seq filtered CpGs (ATAC+, left) and all CpGs (right). “Other” class includes cassettes whose proportion is below 2%.

As shown in Fig. 2A, two major distal cassettes, cassettes 2 and 3, showed correlation with the Basal/non-Basal PAM50 division of the discovery cohort. In contrast, distal cassette 1 (*n* = 22,901 CpGs) and other smaller distal and non-distal cassettes showed no apparent association with these PAM50 groups. A notable feature of distal cassette 1 is a high variance, gradient-like DNA methylation pattern across tumors (Fig. 3A). Moreover, distal cassette 1 showed low overlap with enhancer regions (~23%), similar to other cassettes not linked to the Basal/non-Basal division like distal cassettes 4, 5 and 7, and a relatively high fraction of CpGs overlapping with repetitive sequences (~28%) (Fig. 3B). The overlap of distal cassette CpGs with transcription factor binding sites (TFBSs) also showed distinct patterns: cassettes 1, 4, 5, and 7 showed minimal overlap with TFBSs, mirroring the trend seen in enhancer regions (Fig. 3C). Moreover, CpGs in distal cassette 1 were often located in generally sparse genomic regions (Fig. 3D). Further analyses on potential biological implications of distal cassette 1, with DNA methylation values summarized through the first principal component (PC1), showed no clear difference regarding important tumor characteristics in TNBC such as the PAM50 Basal/non-Basal division, HRD status, TNBC mRNA subtypes, tumor purity, or levels of tumor infiltrating lymphocytes (TILs) (Fig. 3E). Concordantly, when selecting CpGs through supervised methods, such as differential methylation analysis, CpGs included in these specific cassettes were rarely selected (Supplementary Fig. 5). Taken together, both genomic and biological features suggest that distal cassette 1 represents a potential source of high variance, largely non-informative noise rather than biologically relevant CpGs in the context of TNBC.

Importantly, distal cassette 1 could not be removed from the data using variance-based filtering (Fig. 3F), even without applying tumor purity correction (Supplementary Fig. 6). In further support of the non-informativeness of a set of highly variant distal CpGs, we found similar results also after applying a lower soft-thresholding value that minimizes the fraction of unclustered CpGs (Supplementary Fig. 7). Again, the largest detected distal cassette showed low overlap with enhancer regions, lower CpG density, reduced overlap with TFBSs and could not be excluded by variance-based filtering on adjusted or unadjusted beta values (Supplementary Fig. 8). Consequently, if not addressed, less informative, high variance cassettes like distal cassette 1 may influence unsupervised DNA methylation analyses. However, applying an alternative filtering strategy based on chromatin accessibility could successfully remove noise-associated cassettes (Fig. 3G). This approach was based on the overlap of CpGs with ATAC-seq peaks (indicating regions of open, accessible chromatin) from Corces et al.^33^ that have a distinctive distribution per CpG context (Table 2). Specifically, the original distal cassette 1, as well as other cassettes with low overlap with TFBSs and enhancer regions, were removed, while approximately 50% of CpGs in cassettes 2 and 3 were retained (Fig. 3H). Interestingly, the new cassettes derived from ATAC-seq filtered CpGs had clear counterparts among the original cassettes enriched for TFBSs and enhancer regions (Fig. 3I). A similar result was observed when using a lower soft-thresholding value (Supplementary Fig. 9). To explore whether the identified distal cassette patterns were only a feature of TNBC, representing a limited proportion of all breast cancers, we analyzed the methylation patterns of the seven largest distal cassettes in 645 TCGA breast cancers of all molecular and clinical subtypes. Importantly, a similar apparent noise pattern of distal cassette 1, 4, 5, and 7 was observed in all PAM50 subtypes, whereas distal cassette 2 and 3 strongly aligned with a Basal/non-Basal division of the cohort, irrespective of ER, PR, and HER2 status (Supplementary Fig. 10). Together, this highlights the biological relevance and consistency of the identified patterns also in a broader breast cancer setting.

**Table 2:** Distribution of CpGs from the discovery cohort overlapping with ATAC-seq peaks per CpG context

	All	Promoter	Proximal	Distal
ATAC−	205,799	44,722	96,285	394,339
ATAC+	535,346	80,567	44,531	80,701

ATAC+ overlap with Corces et al. ATAC-seq peaks, and ATAC− have no overlap.

### Specific promoter CpG cassettes correlate with TIME status in TNBC

**Fig. 4:**
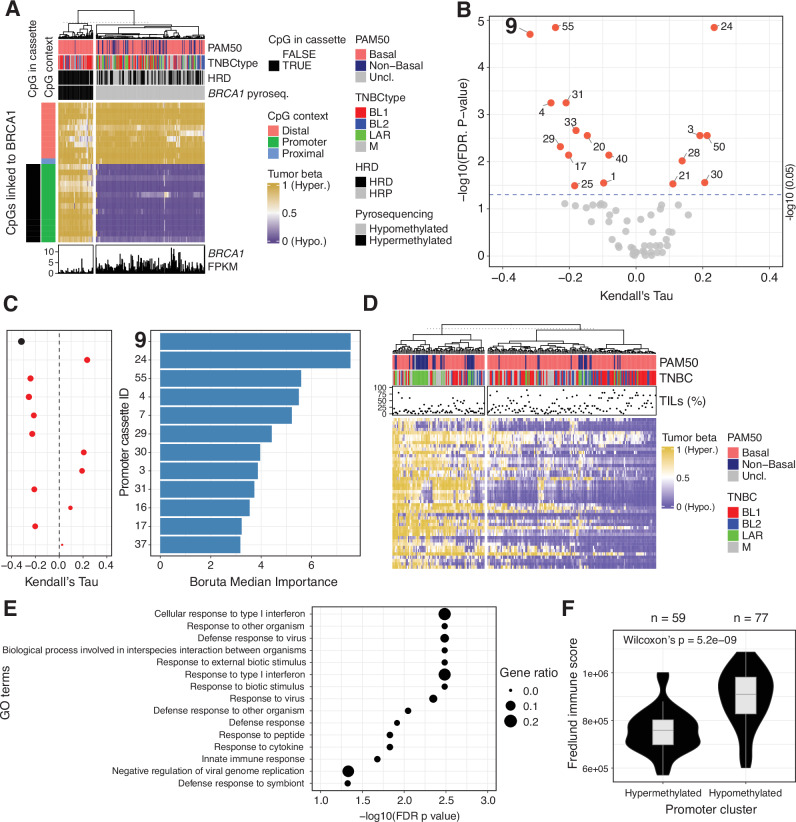
Detection of small promoter cassettes linked to immune infiltration. **A** Association of *BRCA1* promoter hypermethylation with promoter cassette 34. The row annotations represent, from left to right, CpGs in promoter cassette 34 and CpG context. **B** Association between PC1 of promoter cassettes and tumor-infiltrating lymphocytes (TILs). The *x*-axis corresponds to the Kendall correlation coefficient, and the *y*-axis to transformed Benjamini–Hochberg adjusted Wilcoxon *p* values when splitting samples based on methylation patterns using k-means clustering and *k* = 2. Cassette 9 highlighted. **C** Promoter cassettes selected by the Boruta algorithm, their Kendall correlation value with TILs, and median importance from the random forest runs. Numbers on the *y*-axis represent cassette identifiers. **D** DNA methylation beta values of CpGs in promoter cassette 9 (rows) per tumor (columns). Tumors are grouped using k-means clustering and *k* = 2 on the detected cassette’s beta values. **E** Gene Ontology (GO) enrichment of genes associated with the 46 CpGs in promoter cassette 9. **F** Immune metagene rank scores versus hypermethylated and hypomethylated sample groups defined by beta values of CpGs in promoter cassette 9 in the SCAN-B validation cohort. Significance was assessed using Wilcoxon’s test. Sample groups are defined in the same way as in (**D**).

After analyzing the major cassettes reflecting broad biological patterns in TNBC, we focused on smaller cassettes that could be involved in the molecular heterogeneity observed within TNBC. We restricted the analysis to promoter CpG cassettes to facilitate a more direct biological interpretation. As a validation step, we first investigated whether known promoter methylation patterns, such as *BRCA1* promoter hypermethylation was identified by our cassette approach. Indeed, we identified a cassette (promoter cassette 34, *n* = 19 CpGs) including only CpGs associated with *BRCA1* that, on a sample level, showed excellent agreement with both an HRD phenotype and pyrosequencing-based *BRCA1* methylation status (representing an orthogonal methylation analysis method), consistent with previous studies^3,27^ (Fig. 4A).

We next focused on epigenetic mechanisms potentially associated with TIME composition, considering the established significance of the TIME for prognosis and treatment prediction in TNBC^34^. A correlation analysis between PC1 of the 61 promoter cassettes and TIL estimates (acting as a proxy for TIME status) identified a series of cassettes (*n* = 17) that were linked to significant changes in TILs when tumors were split into two groups based on cassette methylation patterns (Fig. 4B). To prioritize cassettes for further study, we used the random forest-based Boruta feature selection algorithm identifying 12 promoter cassettes associated with TIL variation (Fig. 4C, Supplementary Table 4). Among them, we focused on promoter cassette 9 (*n* = 46 CpGs) (Fig. 4D), representing the cassette with the highest absolute Kendall correlation to TILs and Boruta median importance. Sixteen genes were associated with CpGs in promoter cassette 9 (*GBP4, RSAD2, RTP4, SAMD9L, AGBL3, CYREN, TMEM140, CD274, LIPA, BATF2, CARD16, OAS2, XAF1, ZBP1, APOL3*, and *APOL1*) and showed enrichment for immune response terms, particularly innate and antiviral processes (Fig. 4E). Importantly, a similar methylation pattern for cassette 9 CpGs was also observed in the independent SCAN-B validation cohort (Supplementary Fig. 11), where tumors with a hypermethylated pattern showed significantly lower rank scores of a gene expression-based immune response metagene^35^ (Fig. 4F). Finally, a similar link between the methylation pattern for cassette 9 CpGs and TIME status was also observed for TNBCs in the larger 645-sample general TCGA breast cancer cohort, with hypomethylation being associated with increased immune infiltration (Supplementary Fig. 12).

### Features of a promoter CpG cassette associated with TIME status in TNBC

**Fig. 5:**
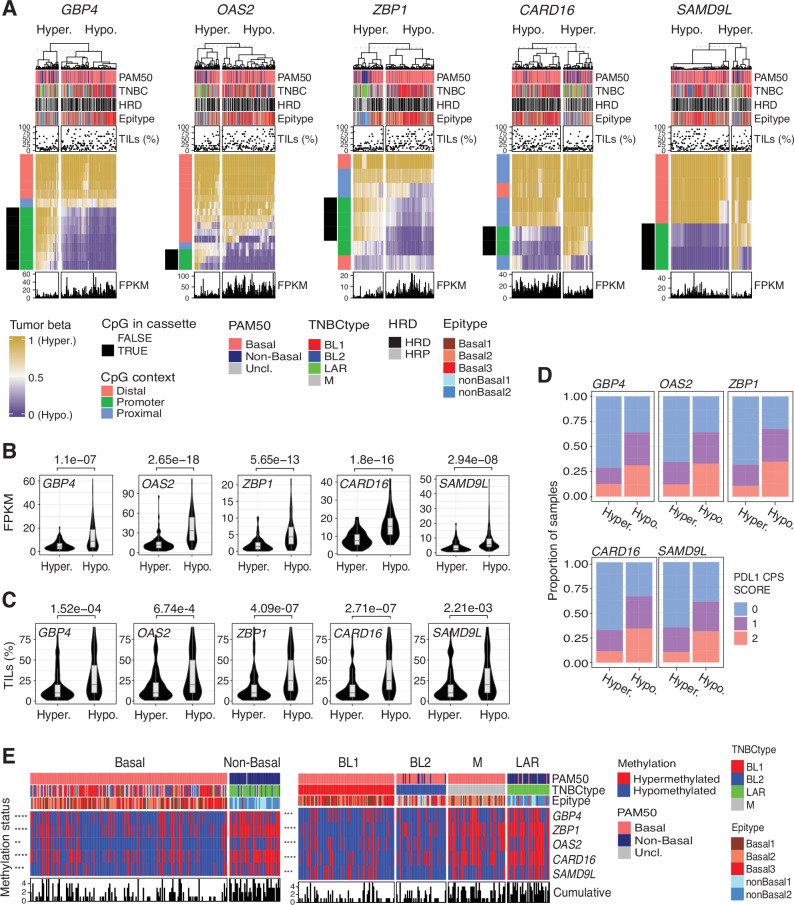
The genes involved in promoter cassette 9 are associated with immune infiltration and show subtype-specific methylation patterns. **A** Methylation state of CpGs overlapping with selected genes, with CpGs included in promoter cassette 9. The samples are grouped using k-means clustering and *k* = 2 on overlapping CpGs’ adjusted beta values for each gene. FPKM gene expression per sample for each of the genes is detailed in the bottom annotation. The row annotations represent, from left to right, the CpGs belonging to promoter cassette 9 (CpG in cassette) and CpG context. **B** Association between observed methylation patterns per gene and FPKM gene expression. The *p* values displayed were obtained using Wilcoxon’s test and adjusted using the Benjamini–Hochberg method. **C** Association between observed methylation patterns per gene and tumor-infiltrating lymphocytes (TILs). The *p* values displayed were obtained using Wilcoxon’s test and adjusted using the Benjamini–Hochberg method. **D** Methylation state per gene versus PDL1-CPS classes. **E** Co-occurrence of the methylation patterns detected per gene in (**A**) (rows) for tumors (columns) of PAM50 Basal/non-Basal (left) and Lehmann TNBCtype (right) TNBC subtypes. Panels below the heatmaps show how many of the five genes have the same methylation status for a sample. The *p* value significance displayed on the left was obtained using Chi-square tests and adjusted using the Benjamini–Hochberg method.

To understand the biological implications of promoter cassette 9 in more depth, we focused on its associated genes with more than one mapped CpG to avoid false positives, leaving 9 genes: *GBP4, SAMD9L, RTP4, CYREN, TMEM140, CARD16, OAS2, XAF1*, and *ZBP1*. The pool of genes was further reduced by excluding genes without a statistically significant association of methylation status with gene expression and TIL levels following Bonferroni correction after their binarization in hyper- and hypomethylated groups for each gene. This left five genes for further analyses: *GBP4*, *OAS2*, *CARD16*, *ZBP1*, and *SAMD9L*, which all showed two distinctive methylation states (hypermethylated and hypomethylated) among the analyzed samples linked to statistically significant differences in gene expression (Fig. 5A, B). The specific DNA methylation and expression patterns per gene were also confirmed in the SCAN-B validation cohort (Supplementary Fig. 13) and in the TNBCs from the general TCGA breast cancer validation cohort (Supplementary Fig. 14A, B). In line with cassette 9’s association with TIL levels, grouping samples by the methylation status of the five genes also showed significant associations with both TIL levels and in situ PDL1-CPS scores, a measure of PDL1 expression in malignant and non-malignant cells (Fig. 5C, D). Specifically, hypermethylated tumors showed a more immune cold TIME, exemplified by lower TIL levels and PDL1-CPS scores. A similar trend of association between the methylation status and TILs was obtained in the TNBCs from the general TCGA breast cancer validation cohort (Supplementary Fig. 14C). Additionally, the methylation states of the identified genes were generally linked to higher infiltration of specific immune cell types such as B cells and cytotoxic T cells, measured through both MethylCIBERSORT immune cell fractions and in situ immune cell counts, with higher infiltration in the hypomethylated samples (Supplementary Fig. 15). Next, we analyzed the co-occurrence of the methylation patterns for the five genes in different TNBC subtypes, finding a high co-occurrence of gene hypermethylation in tumors with a non-Basal PAM50 phenotype and/or Luminal Androgen Receptor and Mesenchymal Lehmann TNBC subtypes (Fig. 5E). In support of this finding, the expression patterns of the five genes were also highly correlated across tumors in the discovery cohort (Supplementary Fig. 16). Similar methylation co-occurrence patterns were observed in the SCAN-B validation cohort (Supplementary Fig. 17). Finally, in the discovery cohort, a trend of higher cumulative hypermethylation status of the five genes with lower immune response metagene rank scores and TILs was observed (Supplementary Fig. 18A). In contrast, an opposite positive trend was observed for rank scores of a gene expression stroma metagene^35^ and fibroblast fraction estimates by MethylCIBERSORT (Supplementary Fig. 18B), together highlighting the potential association of the methylation status with the composition of the tumor microenvironment.

### Characterization of the promoter CpG cassette associated with TIME status across breast cancer subtypes

**Fig. 6:**
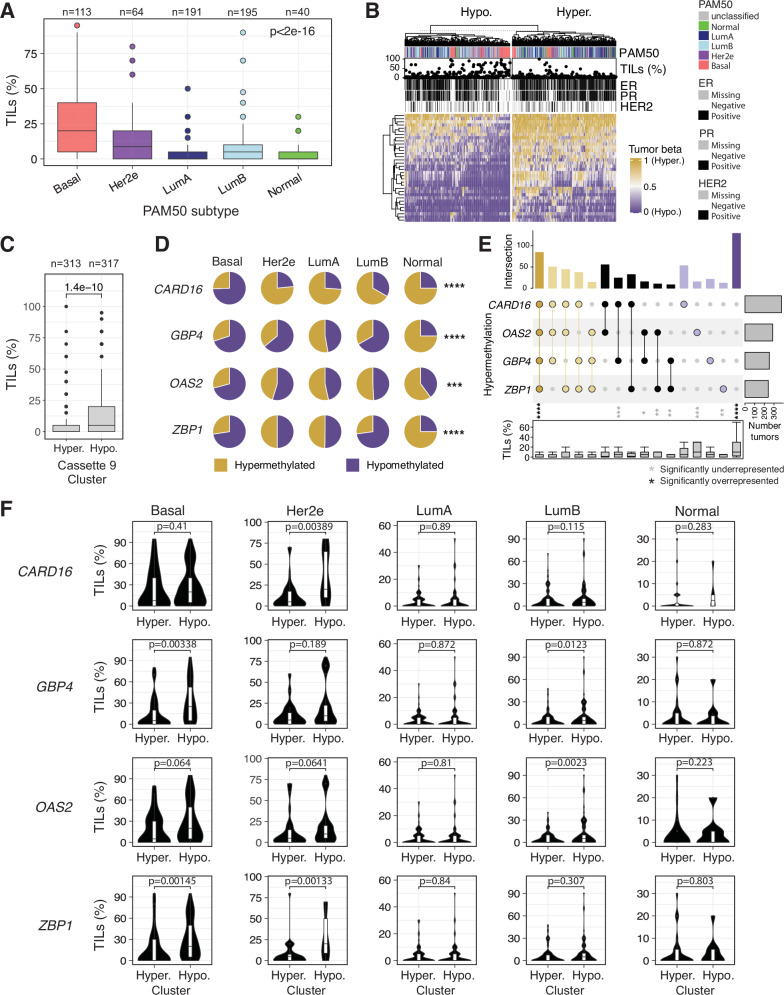
Characterization of TIME-associated CpG cassette and associated genes across breast cancer subtypes. **A** Distribution of TILs across PAM50 breast cancer subtypes in the TCGA breast cancer cohort. Statistical significance was assessed using the Kruskal-Wallis test. Her2e = HER2-enriched. **B** DNA methylation beta values of available CpGs in promoter cassette 9 (rows) across TCGA-BRCA tumors (columns). Tumors are grouped using k-means clustering and *k* = 2 on the detected cassette’s beta values. **C** Association between promoter cassette 9 and immune infiltration in breast cancer. Samples were clustered as performed in (**B**). Statistical significance was assessed using Wilcoxon’s test. **D** Distribution of methylation patterns per gene across breast cancer subtypes. Tumors are grouped using k-means clustering and *k* = 2 on the CpGs overlapping with the genes of interest. Statistical significance was evaluated using the Chi-squared test and adjusted using the Benjamini–Hochberg method. **E** UpSet diagram of shared methylation status per gene across samples. The bottom annotation indicates measured TIL levels for each combination of methylation patterns. Statistical significance was evaluated using two-sided binomial tests with Benjamini–Hochberg correction. **F** Association of TIL levels with methylation status of the detected genes (rows) per breast cancer PAM50 subtype (columns). Clusters were defined as performed in (**D**). Statistical significance was assessed using Wilcoxon’s test and adjusted per gene using the Benjamini–Hochberg method.

Aiming to characterize the role of the identified CpG cassette and genes in a more general breast cancer population, we analyzed their DNA methylation patterns, gene expression, and association with immune infiltration in the 645-sample TCGA breast cancer validation cohort. First, we assessed the differences in immune infiltration per PAM50 breast cancer subtype, observing the highest infiltration in the Basal subtype, followed by HER2-enriched tumors. In contrast, Luminal B and particularly Luminal A and Normal-like samples showed the lowest TIL levels (Fig. 6A). Next, we evaluated whether the detected promoter cassette 9 in TNBC was still present in the complete breast cancer population. Although the structure of the cassette was less binary than in TNBC (Fig. 6B), clustering on the CpGs included in the cassette still revealed a strong association between general hypomethylation and increased immune infiltration (Fig. 6C).

Next, we analyzed whether *GBP4*, *OAS2*, *ZBP1*, and *CARD16* expression was associated with promoter cassette 9 in the TCGA cohort. *SAMD9L* was excluded due to a lack of CpG coverage in the available Infinium 450K methylation data. After grouping samples based on the methylation status of the CpGs overlapping with the respective gene, similar hyper- and hypomethylation patterns to the ones observed in TNBC were identified, which, consistent with TNBC observations, were associated with gene expression levels across all PAM50 subtypes (Supplementary Fig. 19). Moreover, a generally higher fraction of hypermethylated samples per gene was observed in the non-Basal subtypes (Fig. 6D), along with a general co-occurrence of hypermethylation or hypomethylation phenotypes among the genes consistent with TNBC findings, with the co-hypermethylated samples showing generally the lowest TIL levels (Fig. 6E). Finally, we evaluated the association of the DNA methylation status of each gene with immune infiltration within the PAM50 subtypes. In Basal, HER2-enriched, and Luminal B tumors, hypomethylation of the genes was generally associated with higher TILs levels, whereas no difference was identified in Luminal A and Normal-like tumors, likely due to their overall lower immune infiltration (Fig. 6F).

### Methylation and expression patterns of promoter cassette 9-associated genes in normal breast cells

**Fig. 7:**
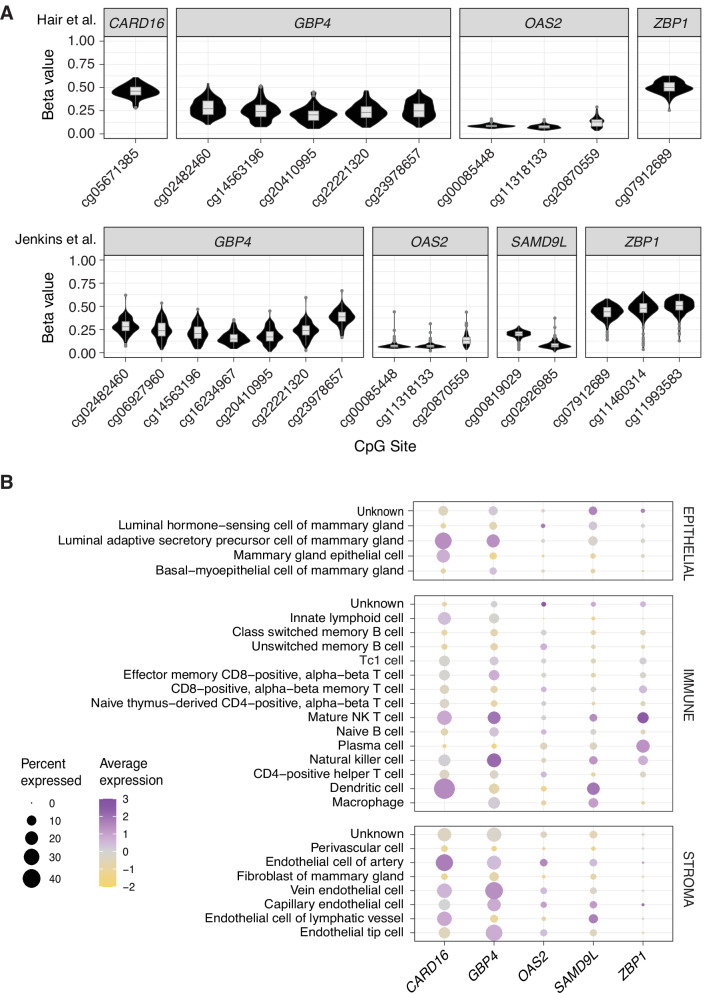
DNA methylation and gene expression of five genes of interest in normal breast tissue. **A** Methylation state of CpGs in promoter cassette 9 affecting specific genes in 96 normal breast tissue specimens from Hair et al.^57^ (top) and in 113 breast tissue samples from Jenkins et al.^58^ (bottom). The CpGs not included here were not available in the used datasets due to different methylation array versions or CpG filtering strategies. **B** Expression of the five genes in normal breast tissue using scRNA-seq data. Cells were grouped based on the assigned cell types in Reed et al.^60^. The average expression, displayed as the color of the dots, represents the average scaled expression of cells belonging to each group. Values were scaled independently for epithelial, immune, and stromal cells. The proportion of cells expressing a gene is represented by the dot size.

To investigate the potential tumor extrinsic expression patterns of the five genes, we analyzed the DNA methylation levels of CpGs in promoter cassette 9 and the expression of the five associated genes in normal breast cells using a combination of reported DNA methylation and single-cell RNA-sequencing (scRNA-seq) data. We observed different methylation states of cassette 9 CpGs per gene in two different normal breast tissue cohorts (Fig. 7A). Three genes, *OAS2*, *GBP4*, and *SAMD9L*, exhibited apparent CpG hypomethylation phenotypes in the datasets analyzed, although the latter two showed less extreme values. CpGs for *CARD16* and *ZBP1* showed beta values around 0.5, indicating a potential mixture of cells with different methylation states in the analyzed bulk tissue. *SAMD9L* and *CARD16* could not be analyzed in both datasets due to a lack of CpGs overlapping with the genes’ promoter regions in the public data. The methylation status of the genes of interest was also assessed in specific immune cell populations using data from flow cytometry-sorted blood samples, revealing distinct hypomethylation for all CpGs in all genes (*SAMD9L* excluded due to lack of CpG coverage, Supplementary Fig. 20).

Next, we analyzed gene expression of the five genes in normal tissue using scRNA-seq data, observing heterogeneous expression in epithelial, immune, and stromal cells (Fig. 7B). In epithelial cells, we observed expression of *CARD16* and *GBP4* in specific subtypes (luminal adaptive secretory precursor cell of mammary gland and mammary gland epithelial cells), while the expression measured for the remaining genes was generally low. In immune cells, the expression was equally heterogeneous: *CARD16* was mainly expressed in dendritic cells and mature natural killer (NK) cells, *GBP4* mostly in different NK cell populations, *SAMD9L* in dendritic cells and macrophages, and *ZBP1* in different NK cell populations and plasma cells. *OAS2*, on the other hand, showed low expression levels in all immune cell types. Finally, in stromal cells, *GBP4* and *CARD16* were expressed in most cell types except fibroblasts of the mammary gland and perivascular cells. *OAS2* and *SAMD9L* showed lower expression, particularly focused on different endothelial cell subtypes, while *ZBP1* showed little expression in any stromal cell type. Taken together, these analyses demonstrate a heterogeneous expression of the five genes in different normal breast and immune cell types, combined with a typically hypomethylated promoter pattern, especially in immune cell types.

### Tumor-intrinsic expression of promoter cassette 9-associated genes

**Fig. 8:**
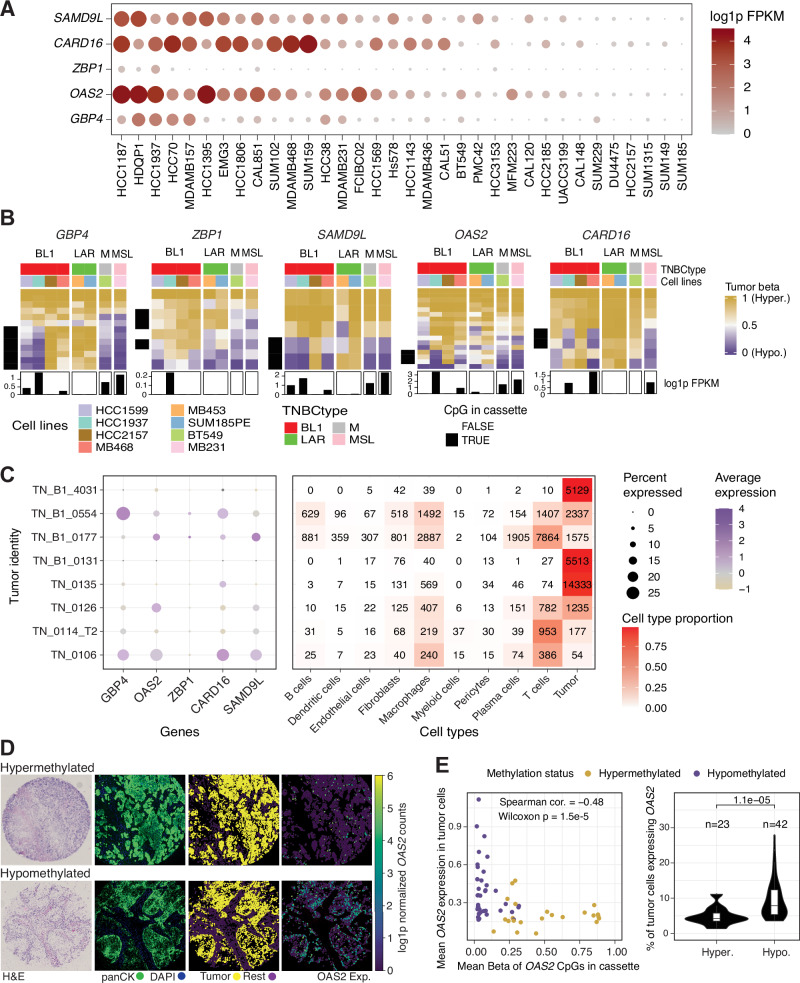
Promoter cassette 9 genes are intrinsically expressed by tumor cells and correlated with tumor-intrinsic methylation patterns. **A** Bulk RNA-sequencing expression of *SAMD9L*, *CARD16*, *ZBP1*, *OAS2*, and *GBP4* in 34 TNBC cell lines. The color and size of the dots represent log1p-transformed FPKM values. **B** DNA methylation of CpGs (rows) connected to the same five genes and their log1p FPKM gene expression (bottom annotation) in eight TNBC cell lines (columns) with matched data. The row annotation represents the CpGs belonging to promoter cassette 9. **C** scRNA-seq gene expression of the five genes in tumor cells from eight TNBC tumor specimens, as well as cell counts of malignant and non-malignant cells. For gene expression in tumor cells, the average expression represents the average scaled expression of tumor cells belonging to each sample. For cell type counts, the color of the tile plot in the right represents the proportion of each cell type per sample, and the number displayed represents the raw cell counts. Cell types were obtained from Chen et al.^62^. **D** Example of tissue microarray cores from one hypomethylated (PD35993a) and one hypermethylated (PD31097a) tumor used in the in situ assessment of *OAS2* expression in tumor cells by the Nanostring CosMx spatial transcriptomics platform. The images from left to right represent: standard H&E staining, immunofluorescence image showing panCK (used as a tumor marker) and DAPI stainings from CosMX analysis, Tumor/Rest masks created per cell based on panCK intensity, and normalized and log1p-transformed *OAS2* expression per cell. **E** Analysis of *OAS2* promoter methylation and expression in tumor cells. Left: Scatter plot showing the relationship between the mean beta value of the *OAS2* promoter CpGs included in cassette 9 and mean OAS2 expression in tumor cells; points are colored according to methylation status (hypomethylated vs hypermethylated), as defined in Fig. 5A. The displayed *p* value corresponds to the comparison of expression vs methylation groups. Right: Violin plot showing the proportion of tumor cells expressing *OAS2* stratified by methylation status. Gene expression values were normalized and log1p-transformed. *N* denotes the number of unique samples. *P* values were calculated using Wilcoxon’s rank-sum test.

To investigate the tumor-intrinsic expression of the five genes associated with promoter cassette 9, we first analyzed bulk gene expression data from 34 TNBC cell lines, representing a tumor microenvironment-free context. We identified tumor-intrinsic expression of *SAMD9L*, *CARD16*, *OAS2*, and *GBP4* in some cell lines, while only very low to no expression of *ZBP1* overall (Fig. 8A). Expression of the five genes was correlated across the 34 cell lines (Supplementary Fig. 21), in line with findings in the discovery cohort. Additionally, in eight previously reported TNBC cell lines with matched RNA-sequencing data and Illumina EPIC DNA methylation, we observed concordance between the methylation state of the promoter CpGs and gene expression levels, i.e., higher expression with CpG hypomethylation (Fig. 8B). Together, these findings support a hypothesis of tumor-intrinsic methylation patterns and expression of the genes by tumor cells. Furthermore, scRNA-seq from 19 TNBC cell lines confirmed tumor-intrinsic expression of the genes in a subset of cell lines, while at the same time revealing substantial cell heterogeneity in expressing cell lines, i.e., cell lines with gene expression comprised both expressing and non-expressing cancer cells (Supplementary Fig. 22).

To substantiate the TNBC cell line observations in actual tumor tissue, we evaluated the tumor-intrinsic expression of the five genes in tumor tissue using scRNA-seq data from eight TNBC patients. Despite the small sample set and substantially different expression levels, we observed co-expression of the identified genes in different samples and a trend that tumors with expression of the genes tended to have an activated TIME, determined as higher counts of immune cells in the scRNA-seq data (Fig. 8C). To further assess the tumor-intrinsic expression of the five genes in situ we analyzed tissue microarray (TMA) cores from 65 tumors in the discovery cohort by spatial transcriptomics using the Nanostring CosMX platform. Focusing on *OAS2* (due to measured expression levels and CosMX gene panel content), we detected a clear anticorrelation between promoter methylation and expression in tumor cells (Spearman correlation = −0.48) and statistically significantly higher mean *OAS2* expression and a higher proportion of tumor cells expressing *OAS2* in tumors classified as hypomethylated by the DNA methylation analysis shown in Fig. 5A (Fig. 8D, E). Importantly, these results connect the in vitro observations with similar findings in primary early-stage treatment naïve tumor tissue, i.e., apparent tumor-intrinsic expression in a subfraction of tumor cells correlating with a tumor-intrinsic hypomethylation status of the promoter region.

## Discussion

In this work, we aimed to characterize methylation dynamics in cancer by investigating the variance structure of bulk tumor DNA methylation data based on the identification of groups of highly correlated CpGs, termed CpG cassettes. In our model system, TNBC, this approach identified CpGs with co-occurring methylation patterns linked to different intrinsic tumor processes and pathways, gene inactivation, and correlation to TIME status, but it also demonstrated the presence of high variance DNA methylation, apparently not biologically relevant for TNBC, that may influence downstream analyses. Moreover, our study further supports that tumor purity adjustment methods can reduce the confounding effects caused by non-malignant cells, thereby increasing the correlation between CpGs with tumor-intrinsic methylation patterns. Together, the current study exemplifies and highlights the utility of network-based analyses of DNA methylation data.

TNBC is a clinical subgroup of breast cancer with two previously reported main methylation patterns linked to a Basal/non-Basal division^21^. Our approach successfully identified those patterns regardless of the CpG context (promoter, proximal, or distal), demonstrating that even small groups of correlated CpGs (cassettes) can accurately predict this division. Notably, our work also revealed that not all high variance methylation patterns appear biologically relevant in the context of the disease in question, an important consideration for unsupervised analysis of DNA methylation data. For instance, the largest detected distal CpG cassette (distal cassette 1) did not show any association with tumor features such as tumor subtype, tumor purity, or TIME status, and the included CpGs showed a low overlap with enhancer regions and transcription factor binding sites, in contrast to the largest promoter and proximal cassettes. Together, these observations suggest that this distal cassette may reflect stochastic noise rather than biologically relevant patterns. Similar observations were made for other distal cassettes as well, e.g., distal CpG cassettes 4, 5, and 7. It is possible that these biologically non-relevant CpG cassettes may be related to assay probe design or to their location in typically non-functional genomic regions. Still, it should be noted that these CpGs are present in both the EPIC and Infinium 450K platforms despite comprehensive data pre-processing and filtering. Moreover, our analysis of the general TCGA breast cancer cohort also demonstrates that these patterns are not specific to TNBC, but instead apply to all breast cancer subtypes, highlighting the generalizability of our findings. It is likely that similar DNA methylation patterns are also present in data from other malignancies. An important observation regarding these non-relevant cassettes was that they could not be removed using variance-based CpG filtering, a common feature selection step before unsupervised analysis. In contrast, supervised analyses appeared less affected by these specific CpGs. Taken together, these non-biological patterns may represent a significant source of bias in unsupervised analyses if not properly addressed, particularly in cohorts without strong apparent DNA methylation states (like the Basal/non-Basal division in TNBC). Nevertheless, a more functionally oriented strategy for filtering CpGs based on chromatin accessibility was able to remove these high variance non-informative cassettes, while retaining the patterns detected in the biologically relevant cassettes that were enriched for functionally active methylation regions. Based on the availability of large-scale ATAC-seq tumor data for different malignancies (e.g., from Corces et al.^33^), our observations support the use of chromatin accessibility-based filtering approaches as an important filtering step when analyzing DNA methylation data, particularly when focusing on distal CpGs.

Beyond the Basal/non-Basal DNA methylation patterns in TNBC, illustrated by several CpG cassettes irrespective of CpG context, our approach also detected cassettes that reflected other tumor-specific patterns like hypermethylation of the *BRCA1* tumor suppressor gene, representing alterations that may at least partially help to explain the molecular heterogeneity within the disease. In a focused exploration of promoter CpG cassettes, we also identified tumor methylation patterns correlated with TIME status in TNBC. The limitation of this analysis to promoter cassettes was due to a more straightforward association of a CpG with a gene when located in a promoter region, and it should be acknowledged that careful analysis of both proximal and distal CpG methylation patterns may reveal additional findings. Supporting the latter, one of the previously reported TNBC DNA methylation epitypes based on tumor purity-adjusted and ATAC-seq filtered distal CpGs showed a notably immune warm phenotype (Basal3)^21^. Among the identified promoter CpG cassettes correlating with TIL levels, we focused on the largest one, promoter cassette 9, and five of the associated genes involved in innate immune response: *GBP4*, *OAS2*, *CARD16*, *ZBP1*, and *SAMD9L*. Promoter hypermethylation in these genes was mainly identified in non-Basal tumors and tumors with a Luminal Androgen Receptor or Mesenchymal-like TNBC mRNA subtype, suggesting an association with tumor-specific phenotypes to be further explored. Both Luminal Androgen Receptor and Mesenchymal-like TNBC subtypes have been described as the more immune-cold subtypes among TNBCs, consistent with our findings^36^. Importantly, further analyses involving cancer cell lines, scRNA-seq, and spatial transcriptomics data of both malignant and non-malignant breast cells supported that the methylation and associated gene expression patterns are likely tumor-intrinsic and not explicitly driven by contaminations from normal or immune cells in the tumor microenvironment.

The five genes connected to promoter CpG cassette 9 are all involved in innate immune processes and have, in some cases, been associated with antitumor immune response. *GBP4* is a GTPase belonging to the Guanylate Binding Protein family, which are central regulators of the innate immune response^37^ and are involved in relevant innate immune processes such as the formation of the inflammasome^38^. Specifically, *GBP4* has been widely identified as relevant for tumor biology in different cancer types; it has been recognized as a pan-cancer marker of immune warm tumors^39^, its promoter’s hypomethylation and expression have been linked to T cell infiltration and recruitment in vitro in pancreatic cancer^40^, as well as a good prognosis biomarker in intrahepatic cholangiocarcinoma, where it was associated with higher activity of various innate immune pathways^41^. *OAS2* is an innate immune protein involved in antiviral response^42^. *ZBP1*, on the other hand, is an immune sensor of Z conformation nucleic acids and participates in the immune response against different pathogens^43^. This gene has been involved in the regulation of immune response in cancer through the activation of necroptosis or PANoptosis^44,45^. *SAMD9L* has a clear role promoting antiviral immunity and has been identified as a tumor suppressor^46^. Finally, *CARD16* plays a less clear role in tumors. This protein belongs to the caspase-1 protein subfamily^47^, which is generally involved in proinflammatory mechanisms and the regulation of the inflammasome activation^48^.

Given their tumor-intrinsic expression, potential epigenetic regulation, and functional implications for the innate immune response, the five genes associated with promoter cassette 9 may play an important role in shaping the composition of the TIME in TNBC, but potentially also in other breast cancer subtypes, as suggested by our exploratory analysis of the general TCGA breast cancer cohort. A notable feature of the expression pattern of the five genes is the apparent intra-tumor heterogeneity, with both expressing and non-expressing tumor cells within the same tissue specimen. Interestingly, this heterogeneity mirrors recent findings by us for PDL1 in both TNBC cell lines and in primary tumors of a specific proposed epitype of TNBC (Basal3)^21^. Because we cannot, at this point, reliably deconstruct tumor methylome clonality, particularly in tumor purity-adjusted bulk data due to the nature of the processing, it remains unclear whether the observed heterogeneity in expression is mirrored by a similar heterogeneity in promoter methylation status, or if it is instead determined by microenvironmental signals or stochastic expression. Furthermore, an important limitation of the current study is that causal conclusions cannot be drawn due to its observational nature. It is possible that the observed promoter methylation patterns could reflect downstream effects of immune-related signaling, particularly hypomethylation induced by interferon-γ signaling, as the specific genes are interferon-inducible. Nevertheless, several findings support the hypothesis of a causal relationship between hypermethylation of the detected genes and an “immune cold” TIME. First, the detected hypomethylation pattern is not exclusive to samples with high TIL levels. Some samples with low immune responses measured through TILs show the methylation phenotype, which could indicate that it is not a consequence of immune response-related signaling pathways and that alternative alterations may be more important in some cases. Second, CpGs associated with *OAS2* and *GBP4* are generally hypomethylated in normal breast tissue, implying that the TIME-associated CpG sites may become hypermethylated relative to a hypomethylated baseline, rather than undergoing demethylation. Third, prior analyses have identified a relationship between T-cell recruitment and hypomethylation of *GBP4* promoter CpGs in vitro in pancreatic cancer, suggesting a causal role of hypermethylation in acquiring an immune cold phenotype^40^. Altogether, these observations support a hypothesis that hypermethylation and lack of expression of the identified genes are linked to reduced immune infiltration in TNBC, particularly in specific molecular phenotypes. However, whether these methylation patterns depend on the cell of origin of the tumor or are acquired during tumorigenesis remains unclear and cannot be assessed by the data presented in this work. Additional experimental validation in vitro and in vivo is needed to clarify the role of the detected methylation patterns in tumors and the relevance of gene expression in different normal breast cell types to establish stronger causal relationships and help identify the origin of the observed epigenetic features.

In conclusion, this work outlines a new comprehensive framework to analyze bulk tumor DNA methylation data, combining tumor purity adjustment, functional CpG filtering, stratification by CpG contexts, and identification of highly correlated CpG modules to enhance the identification of tumor-intrinsic DNA methylation patterns and their biological associations. Importantly, this approach extends beyond TNBC and may help to deconstruct DNA methylation patterns and thereby molecular heterogeneity also in other malignancies. In TNBC, this methodology, in combination with additional -omics layers and in situ data, identified CpG cassettes not only associated with the major molecular subtypes of the disease, but also smaller cassettes linked to tumor suppressor gene inactivation. Moreover, it also identified cassettes correlating with TIME status that appeared enriched in distinct molecular phenotypes of TNBC as well as other tumor subgroups, lending support to the broader concept of epigenetic immunoediting, as previously proposed in glioblastoma^49^. Intriguing aspects that remain to be answered with respect to potential epigenetic immunoediting relate to the plasticity of these changes during treatment with, for instance, immune checkpoint inhibitors and whether treatment responses differ depending on the different mechanisms.

## Methods

### Inclusion and ethics statement

Patients were enrolled in the Sweden Cancerome Analysis Network – Breast (SCAN-B) study (ClinicalTrials.gov ID NCT02306096)^50,51^ approved by the Regional Ethical Review Board in Lund, Sweden (registration numbers 2009/658, 2010/383, 2012/58, 2013/459, 2014/521, 2015/277, 2016/541, 2016/742, 2016/944, 2018/267) and the Swedish Ethical Review Authority (registration numbers 2019-01252, 2024-02040-02). All patients provided written informed consent prior to enrollment, including to publish information about sex and age. All analyses were performed in accordance with patient consent and ethical regulations and decisions. Patient gender was not considered as an inclusion or exclusion criterion for the study. This study conformed to the principles of the Helsinki Declaration.

### SCAN-B primary TNBC datasets

This work is based on two TNBC cohorts, used for discovery and validation purposes. Both cohorts belong to the Swedish SCAN-B study, and all analyzed tissue specimens were taken prior to any treatment. In Sweden, the definition of TNBC is a tumor with <10% of cells with immunohistochemistry (IHC) staining for ER and PR (thus including tumors with 1–9% stained cells) and an IHC HER2-staining score of <2, or for patients with IHC 2+, a non-amplified in situ hybridization status. All SCAN-B data for ER, PR, and HER2 status were obtained from clinical routine analyses performed by regional pathology departments and provided through the national Swedish breast cancer quality registry. The discovery cohort consists of clinical, pathological, and whole genome sequencing (WGS) data from a population representative set of 235 TNBC patients originally reported by Staaf et al.^27^. Molecular data layers included DNA methylation data based on Illumina Infinium MethylationEPIC BeadChip arrays obtained from Aine et al.^21^ and RNA-sequencing data summarized as fragments per kilobase million (FPKM) counts obtained from Staaf et al.^52^. Different TNBC sample classifications such as PAM50 subtypes, the four refined Lehmann TNBC subtypes (TNBCtype: BL1, BL2, M, and LAR), DNA methylation epitypes (Basal1, Basal2, Basal3, nonBasal1, and nonBasal2), HRD status (HRD or HR proficient: HRP), and rank-based gene expression scores from eight biological metagene genes (including a stroma and immune response metagene^35^) were obtained from Aine et al.^21^. Additionally, pathology estimates of TILs and PDL1 combined positive score (PDL1-CPS) were collected from Aine et al.^53^ and Sigurjonsdottir et al.^54^, respectively. CPS scores were categorized into three groups as CPS 0 (CPS score <1), CPS 1 (CPS score 1–9), and CPS 2 (CPS score ≥10). Digital cell counts of relevant immune cell type markers such as CD20, CD3, CD8, FOXP3, CD4, CD68, and PDL1 were obtained from Roostee et al.^29^ calculated with the TMArQ software. A detailed description of the inclusion and exclusion criteria of patients in the discovery cohort is available in the original publication^27^. The validation cohort consists of molecular and clinical data from 136 TNBC samples reported by Aine et al.^21^. Molecular data layers included are DNA methylation data based on Illumina Infinium MethylationEPIC BeadChips obtained from Aine et al.^21^ and RNA-sequencing data summarized as FPKM counts obtained from Staaf et al.^52^. Different TNBC sample classifications, such as PAM50 subtypes, Lehmann TNBC subtypes, and DNA methylation epitypes, were obtained from Aine et al.^21^. The composition and characteristics of the discovery and validation cohorts are detailed in Table 1.

Processed and filtered tumor purity-adjusted DNA methylation data^16,17^ in the form of beta values (range 0–1, representing hypomethylated to hypermethylated) of both cohorts, comprising in total 741145 CpGs in the discovery cohort and 717458 CpGs in the validation cohort, were obtained from Aine et al.^21^. Briefly, the tumor purity adjustment of beta values as originally outlined by Staaf and Aine^16^ comprises two main steps: (i) estimation of tumor purities for individual samples, and (ii) subsequent adjustment of beta values per CpG per sample using linear regression models to account for the influence of non-malignant cells. As demonstrated by Sasiain et al.^17^, tumor purity estimates obtained from different algorithms tend to be highly correlated, and moreover, as demonstrated by Staaf and Aine^16^, minor variations in these estimates have little effect on the resulting adjusted beta values. Data regarding CpG characteristics were compiled from different sources. CpG context, chromosome accessibility status based on ATAC-seq, and overlap with repetitive sequences and transcription factor binding sites were obtained from Aine et al.^21^. Overlap of CpGs with enhancer regions was assessed from enhancers annotated in the GeneHancer genomic regulatory element database (v5.24)^55^. CpG density was determined based on the genomic location of each CpG using windows of 5000 base pairs centered on the CpG and calculated as the number of CpGs in the window divided by the total base pairs. Assignment of CpGs to a gene-centric context was performed as outlined in Aine et al.^21^. Briefly, CpGs were defined as promoter (±500 bp centered on gene transcription start site, TSS), proximal (±5 kbp centered on TSS and excluding the promoter window), or distal (>5 kbp from TSS) based on their genomic coordinates (referred to as genic context). For the gene-centric annotations, a consensus transcript model based on GENCODE v27 protein-coding genes matching SCAN-B RNA-sequencing data was built for each gene by collapsing exons. The 5′ most base was assigned as the consensus TSS, and the 3′ most base as the consensus transcription termination site.

### TCGA-BRCA external validation dataset

An external validation cohort of 645 breast cancers, representing all molecular and clinical breast cancer subtypes, from The Cancer Genome Atlas (TCGA, referred to as TCGA-BRCA) with DNA methylation data (Illumina Infinium 450K) and RNA-sequencing data summarized as FPKM values was obtained from Aine et al.^21^. Pathologist estimated TIL values for the samples were obtained from Cha et al.^56^.

### Breast tissue datasets

Molecular data from normal breast tissue and immune cells were obtained from four different sources. Illumina Infinium 450K DNA methylation data from 96 normal breast tissue samples from mastectomies, breast reductions, and prophylactic tissue were obtained from Hair et al.^57^ and deposited in Gene Expression Omnibus (GEO) under accession number GSE67919. Infinium EPIC 850K DNA methylation data from 113 breast tissue samples from reduction mammoplasty (*n* = 108) and tumor adjacent normal tissue (*n* = 5) were obtained from Jenkins et al.^58^, deposited in the GEO under accession number GSE225845. Illumina Infinium 450K DNA methylation profiles for 10 different flow cytometry sorted blood cell types/fractions were obtained from Reinius et al.^59^ deposited in GEO under accession number GSE35069. scRNA-seq data from normal breast tissue, together with cell type annotations, were obtained from Reed et al.^60^ deposited in the CellXGene platform.

### TNBC cell line datasets

Bulk RNA-sequencing data from 34 TNBC cell lines were obtained from Jovanovic et al.^61^ summarized as FPKM data, and deposited under the GEO accession number GSE202770. Eight TNBC cell lines with available RNA-sequencing data, summarized as FPKM values, and Illumina EPIC DNA methylation data were obtained from Aine et al.^21^. Additionally, 19 cell lines with available scRNA-seq data were obtained from Jovanovic et al.^61^ deposited under the GEO accession number GSE202771, and processed as described by Aine et al.^21^. Cell lines were only used to contrast gene-specific promoter methylation and mRNA expression patterns. Processed scRNA-seq data together with cell type annotations from eight TNBC tumor samples were collected from Chen et al.^62^ deposited under the GEO accession number GSE161529.

### Spatial transcriptomics TNBC dataset

Single-cell spatial transcriptomics data from 65 tumors from the discovery cohort were used to assess *OAS2* expression in situ. Spatial transcriptomics analysis was performed using the Nanostring/Bruker CosMx instrument on two tissue microarray (TMA) sections, generally containing two 1mm cores per tumor (114 cores in total), using the Universal Gene Characterisation panel (1000 transcripts). Briefly, TMA sections 4 μm thick were cut and mounted on VWR Superfrost Plus slides, without coverslips. After sectioning, slides were dried at 37 °C overnight at an angle no greater than 45°. Slides were stored at 4 °C with a desiccant bag until analysis. Summarized clinicopathological and molecular characteristics for the 65 tumors are available in Supplementary Table 1. CosMx analysis was performed following the manufacturer’s instructions at the Spatial Biology Facility, Faculty of Life Sciences and Medicine, King’s College, London, UK. The image files were processed using Nanostring/Bruker’s AtoMx platform and the in-house pipeline and quality assurance setup at the Spatial Biology Facility.

### Identification and analysis of CpG cassettes

CpG cassettes were identified using Weighted Gene Correlation Network Analysis (WGCNA) through the WGCNA R package (v1.73)^63^. First, methylation beta values were log transformed into *M* values (*M* = log2(Beta/(1 − Beta))) after capping beta values <0.01 and >0.99 to avoid extreme values. The blockwiseModules() function was applied using Pearson as the correlation measure and the following parameters: networkType = “unsigned,” minModuleSize = 15, maxBlockSize = 10,000, reassignThreshold = 0, deepSplit = 2, and mergeCutHeight = 0.25. Different soft-thresholding beta values (*β*), the parameter that determined the stringency of the clustering approach, were used to run the analyses (5, 6, 7, 8, 10, and 15) and evaluated using the pickSoftThreshold() function. The optimal soft-threshold power was set to 7 using tumor purity-adjusted and not context-stratified methylation data and was kept across runs to maximize consistency. The remaining parameters were set to the default values. The cassette detection was run on *M* values from both tumor purity-adjusted and unadjusted CpG beta values, without CpG context stratification, only accounting for CpGs with variances (measured on beta values) below 0.05. This approach was also applied to both tumor purity-adjusted and unadjusted CpG beta values after CpG context stratification, using 0.05 as the variance threshold for promoter, proximal, and distal cassettes. Additionally, the same approach and the same parameters were used to identify CpG cassettes from distal CpGs filtered based on chromatin accessibility, defined as CpG overlap with breast cancer ATAC-seq peaks from the study by Corces et al.^33^ (see Aine et al.^21^ for details). For each cassette, the first principal component (PC1) of the DNA methylation *M* values was computed as a summary measure. The sign of PC1 was adjusted to ensure it aligned with the original data, so that higher values consistently reflected increased methylation beta values, facilitating interpretability. While the WGCNA analysis was performed on *M* values, all plotting was performed using beta values, as these are intuitively easier to interpret.

To analyze the impact of tumor purity adjustment on the methylation data and to obtain estimates of the cell composition of each sample, the MethylCIBERSORT R package (v0.2.1)^64^ was used to generate mixture files from adjusted and unadjusted methylation data using the Prep.CancerType() function and the reference breast cancer signatures (breast_v2). The obtained data were used to run deconvolution through the CIBERSORTx online tool^65^, using 1000 permutations and disabling quantile normalization.

The stability of the detected CpG cassettes was analyzed on tumor purity-adjusted and CpG context-stratified data. After filtering CpGs based on variance as detailed previously, 20 groups of 100 samples were randomly selected from the discovery cohort, using the R sample() function with probabilities reflecting the original distribution of the DNA methylation epitypes to avoid class imbalance. Cassette detection was then performed as previously described.

The preservation of the identified CpG cassettes in the SCAN-B validation cohort was analyzed using the modulePreservation() function from the WGCNA package using 200 permutations^32^. The Zsummary value, which integrates several cassette stability metrics, was used to evaluate module preservation.

The predictive capacity of each major CpG cassette in calling the PAM50 Basal/non-Basal division was determined through the ARI using the adjustedRandIndex() function from the mclust (v6.1.1) R package. Specifically, for each cassette, the PAM50 Basal/non-Basal tumor subtype classification was compared to the resulting two-sample clusters obtained from hierarchical clustering (using Euclidean distance and Ward.D2 linkage) of CpG methylation data, setting the number of clusters to two. Additionally, cassettes with ARI > 0.55 were used to stratify samples in Basal/non-Basal groups using hierarchical clustering (Euclidean distance, Ward.D2 linkage) to evaluate their predictive capacity.

Gene overrepresentation analysis of the CpGs included in the analyzed cassettes was performed using the missMethyl R package (v1.38.0)^66^, which, among others, adjusts for the varying numbers of CpGs per gene^67^. The gometh() function was used to perform Gene Ontology enrichment with the following parameters: collection = “GO,” array.type = “EPIC,” prior.prob = TRUE, equiv.cpg = TRUE, fract.counts = TRUE, and genomic.features = “ALL.” To account for the CpG context-based stratification, the all.cpg argument was set to include only the CpGs from the corresponding context being analyzed. The same function and parameters were used to perform enrichment of KEGG (Kyoto Encyclopedia of Genes and Genomes) pathways, but setting collection = “KEGG.” Cancer Hallmark enrichment was determined using the gsameth() function with the same parameters as above, but using human hallmarks signatures (v7.1) obtained from the Molecular Signatures Database (MSigDB)^68^ as the collection parameter.

The association of CpG cassettes with gene expression differences was assessed by linking CpGs in individual cassettes to genes using the provided UCSC annotations. For each gene, tumors were divided into two groups based on k-means clustering of CpGs connected to the gene through the kmeans() R function, using centers = 2 and the default parameters, and mRNA expression differences between the two methylation groups were analyzed using Wilcoxon’s test.

The top 1000, 5000, 10,000, 20,000, and 30,000 most variable CpGs were determined in tumor purity-adjusted and unadjusted DNA methylation data, respectively. The proportion of each detected cassette within each CpG context and CpG set was calculated by identifying the CpGs shared between the cassette and the top N groups.

Adjusted DNA methylation data from the discovery cohort were used to identify differentially methylated CpGs per molecular subtype between PAM50 Basal/non-Basal and the Lehmann TNBC subtype division. Differential methylation was tested pairwise between each molecular subtype using Wilcoxon’s test, followed by Bonferroni multiple testing correction. Finally, the overlap of significant CpGs with identified distal CpG cassettes was analyzed.

### Identification and analysis of CpG cassettes associated with immune infiltration

To identify CpG cassettes associated with TILs, we performed an association analysis between cassettes and TIL proportions. First, the association was tested using Kendall’s correlation value between TILs and the PC1 values of the analyzed cassettes. Additionally, aiming to capture the two potential methylation states of CpGs, we defined two methylation groups per cassette by applying k-means clustering on their PC1 values, setting the number of clusters to 2. Next, we compared TIL proportions between these groups using Wilcoxon’s test. To prioritize cassettes relevant for TILs, we used the Boruta algorithm, using PC1 of each CpG cassette as a summary value. Feature selection was performed using the Boruta() function from the Boruta R package (v8.0.0)^69^, setting doTrace = 2, maxRuns = 500, and remaining parameters to default.

Analyses of scRNA-seq data from tumor and normal breast samples were performed using the Seurat R package (v5.3.0)^70^ together with cell type annotations from the original publications. The DotPlot() function with default parameters was used for gene expression analyses.

The CosMx spatial transcriptomics data were analyzed using the Python Squidpy (v1.6.5)^71^ and Scanpy (v1.10.4)^72^ libraries. Cells with a proportion of negative probabilities >4%, <30 transcripts detected, and <30 different genes detected were removed. Transcript counts were then normalized using the normalize_total() function and setting target_sum = 1e4 followed by log1p transformation through the log1p() function. Tumor cells were identified per TMA slide through Gaussian Mixture Modelling applied on the mean panCK intensity detected per cell using the scikit-learn Python library (v1.6.1)^73^.

### Statistical analyses, data handling, and plotting

Data handling, statistical analyses, and plotting were run in R (v4.4.1). Data handling was performed through base R functions, and the dplyr (v1.1.4), tidyr (v1.3.1), and reshape2 (v1.4.4) packages. Pairwise comparisons were performed using two-sided Wilcoxon’s test. Comparisons between three or more groups were performed using the two-sided Kruskal-Wallis test. The Chi-squared statistical test (two-sided) was used for comparisons between categorical variables. The binomial test was used to compare observed and expected events. Benjamini–Hochberg or Bonferroni multiple testing correction was implemented when necessary to control for the expected proportions of false positives using the p.adjust() function, setting the method to “fdr” or “bonferroni,” respectively. Asterisks indicate *p* value significance as follows: * when *p* ≤ 0.05, ** when *p* ≤ 0.01, *** when *p* ≤ 0.001, **** when *p* ≤ 0.0001, and ns when *p* > 0.05. Correlation values were determined using the Spearman and Kendall methods. Plotting was performed using base R functions, ggplot2 (v3.5.2), ComplexHeatmap (v2.20.2), ggsankey (v0.0.99999), corrplot (v0.95), VennDiagram (v1.6.0), and other R packages’ specific plotting functions. Column clustering of every heatmap was performed using Euclidean distance and the Ward.D2 method, while the rows were kept unclustered if not specified otherwise. Boxplot elements correspond to: (i) center line = median, (ii) box limits = upper and lower quartiles, and (iii) whiskers = 1.5× interquartile range.

## Supplementary information

Supplementary Information

Supplementary Files

## Data Availability

For the discovery cohort, used WGS data originally reported by Staaf et al.^27^ are available from [https://data.mendeley.com/datasets/2mn4ctdpxp/3], while used RNA-sequencing data originally reported by Staaf et al.^52^ are available from [https://data.mendeley.com/datasets/yzxtxn4nmd/3]. DNA methylation data used for the discovery cohort were originally reported by Aine et al.^21^ and are deposited in the Gene Expression Omnibus under the GSE148748 and GSE148906 accession numbers. DNA methylation data used for the SCAN-B validation cohort were previously reported in Aine et al.^21^ and it is deposited in the Gene Expression Omnibus (GEO) under accession number GSE290981. RNA-sequencing data from this cohort are available from [https://data.mendeley.com/datasets/yzxtxn4nmd/3]. TILs and PDL1-CPS estimates for the discovery cohort from Aine et al.^53^ and Sigurjonsdottir et al.^54^, respectively, are available in the original publications. TMArQ determined immune cell counts per sample in the discovery cohort by Roostee et al.^29^ are available from the original publication. Sample annotations such as PAM50 subtypes, the four refined Lehmann TNBC subtypes, DNA methylation epitypes, HRD status, and rank-based gene expression scores for both the discovery and validation cohorts were obtained from the publication by Aine et al.^21^. Raw RNA-sequencing data used in this study and generated by Aine et al.^21^ for eight TNBC cell lines are available through the Short Read Archive (SRA) archive at NCBI under BioProject accession PRJNA1189708 and study SRP547133. Corresponding DNA methylation data from Aine et al.^21^ for the eight TNBC cell lines are deposited in the GEO database (accession number GSE282347). The previously reported breast cancer TCGA data used in this study are available from the GDC data portal [https://portal.gdc.cancer.gov]. TIL estimates for the TCGA breast cancer cohort are available from Cha et al.^56^. The previously reported normal breast tissue DNA methylation data from Hair et al.^57^ used in this study are deposited in the Gene Expression Omnibus under the accession number GSE67919. The previously reported breast tissue DNA methylation data from Jenkins et al.^58^ used in this study are deposited in the Gene Expression Omnibus under the accession number GSE225845. The previously reported scRNA-seq data of normal breast tissue from Reed et al.^60^ are deposited in the CellXGene platform and available from [https://cellxgene.cziscience.com/collections/48259aa8-f168-4bf5-b797-af8e88da6637]. The previously reported RNA-sequencing data for TNBC cell lines from Jovanovic et al.^61^ used in this study are deposited in GEO (accession number GSE202770). The previously reported TNBC scRNA-seq data from Chen et al.^62^ used in this study are deposited in GEO (accession number GSE161529). The previously reported sorted immune cell DNA methylation data from Reinius et al.^59^ used in this study are deposited in GEO (accession number GSE35069). The gene enhancer dataset was retrieved from the GeneHancer genomic regulatory element database (v5.24)^55^, and it is available from [https://www.genecards.org/Guide/GeneHancer]. The CosMx spatial transcriptomics data required for the analyses performed in this work are available as Supplementary Data File 5.

## Code availability

The code used for the analyses implemented in this paper is available in the following GitHub repository: https://github.com/StaafLab/methylation_dynamics_tnbc.
