# Supplementary material for: Genome-wide DNA methylation profiling of triple-negative breast cancer uncovers epigenetic biomarkers of tumor identity and the immune microenvironment
Source: NPJ Breast Cancer. 2026 Aug 12;12:105. doi: 10.1038/s41523-026-01030-y (PMC13469125; doi:10.1038/s41523-026-01030-y)
Supplement: Supplementary file 1 [file 41523_2026_1030_MOESM1_ESM.pdf]

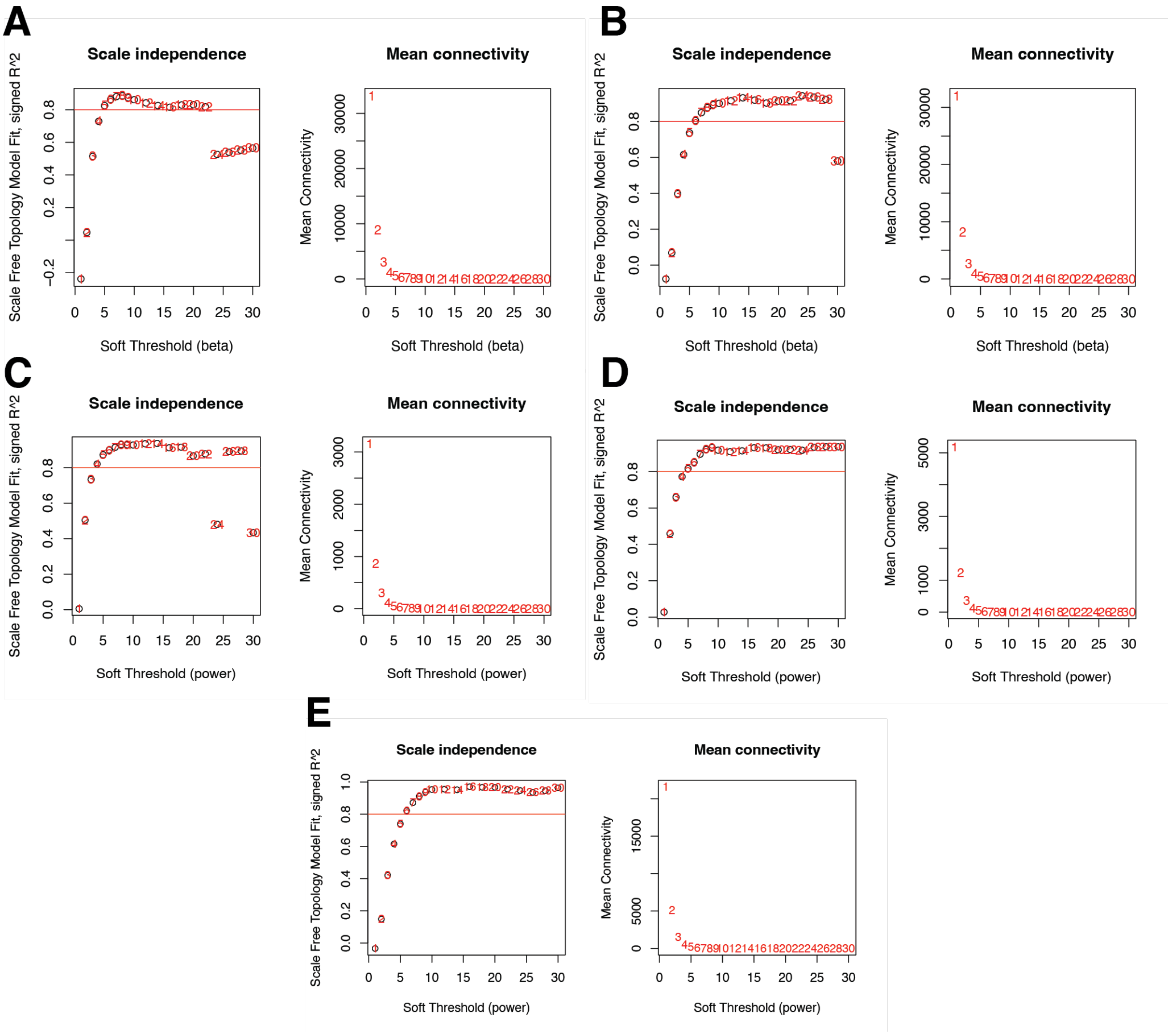

Supplementary Figure 1. Selection of soft thresholding power in WGCNA using the discovery cohort. Soft threshold (β) selection for **A**) all purity-adjusted CpG beta values, **B**) all unadjusted CpG beta values, **C**) promoter purity-adjusted CpG beta values, **D**) proximal purity-adjusted CpG beta values, and **E**) distal purity-adjusted CpG beta values. Low-variance CpGs were excluded to reduce computational load: CpGs with variance<0.05 were removed in A, C, D and E. For panel B, the same number of high-variance CpGs as in A was used. In each case, the left panel shows the scale-free topology model fit versus soft threshold, identifying the saturation point to be between 6 and 10. The right panel shows connectivity versus soft threshold, indicating a sharp decrease when beta increases, reflecting increasing network sparsity. Based on these plots, β=7 was selected for all subsequent WGCNA analyses.

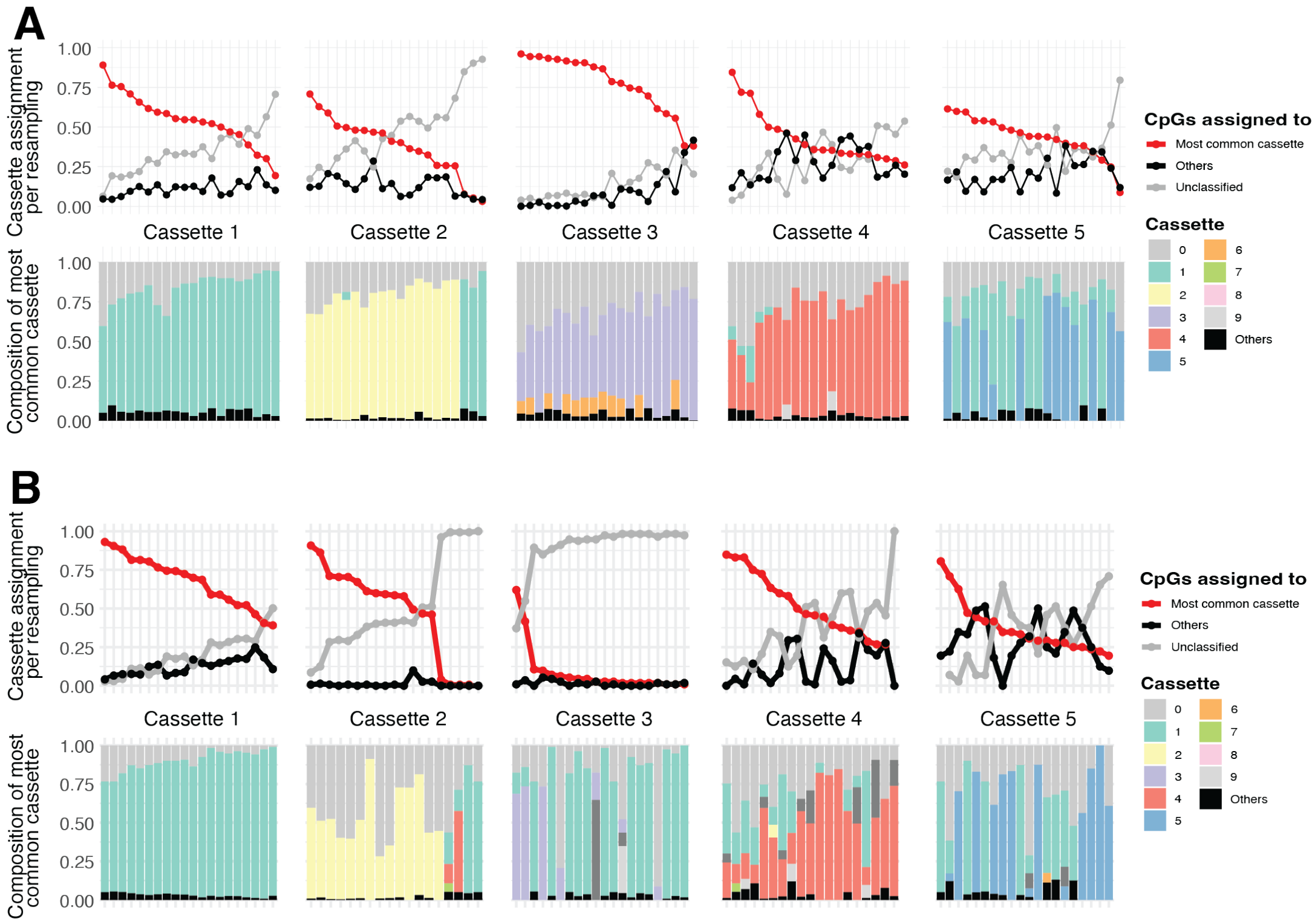

Supplementary Figure 2. Stability analysis of major promoter (A) and proximal (B) cassettes in the discovery cohort. To assess the stability of the detected cassettes, 100 samples were randomly resampled 20 times while preserving proposed DNA methylation epitype proportions from Aine et al. Top row: for each CpG site originally assigned to a given cassette, the proportion of resamplings in which it was assigned to the most common cassette (red), to any cassette (black), or to a cassette other than the most common (gray) is shown. A high red line indicates consistent reassignment to the same cassette across resamplings. Bottom row: proportional cassette assignments for each CpG site across resamplings, with colors representing the cassettes to which CpGs were assigned. Uniform colors within a cassette indicate strong preservation of the original group structure. CpGs from the original proximal and promoter cassettes generally clustered together across resamplings, forming cassettes largely composed of CpGs from the same original cassette. Nevertheless, some cassettes, such as promoter cassette 3 was kept mostly unclustered in most of the resamplings, indicating a weaker correlation structure, and other cassettes such as promoter and proximal cassette 5 appeared often co-clustered with promoter and proximal cassettes 1, respectively.

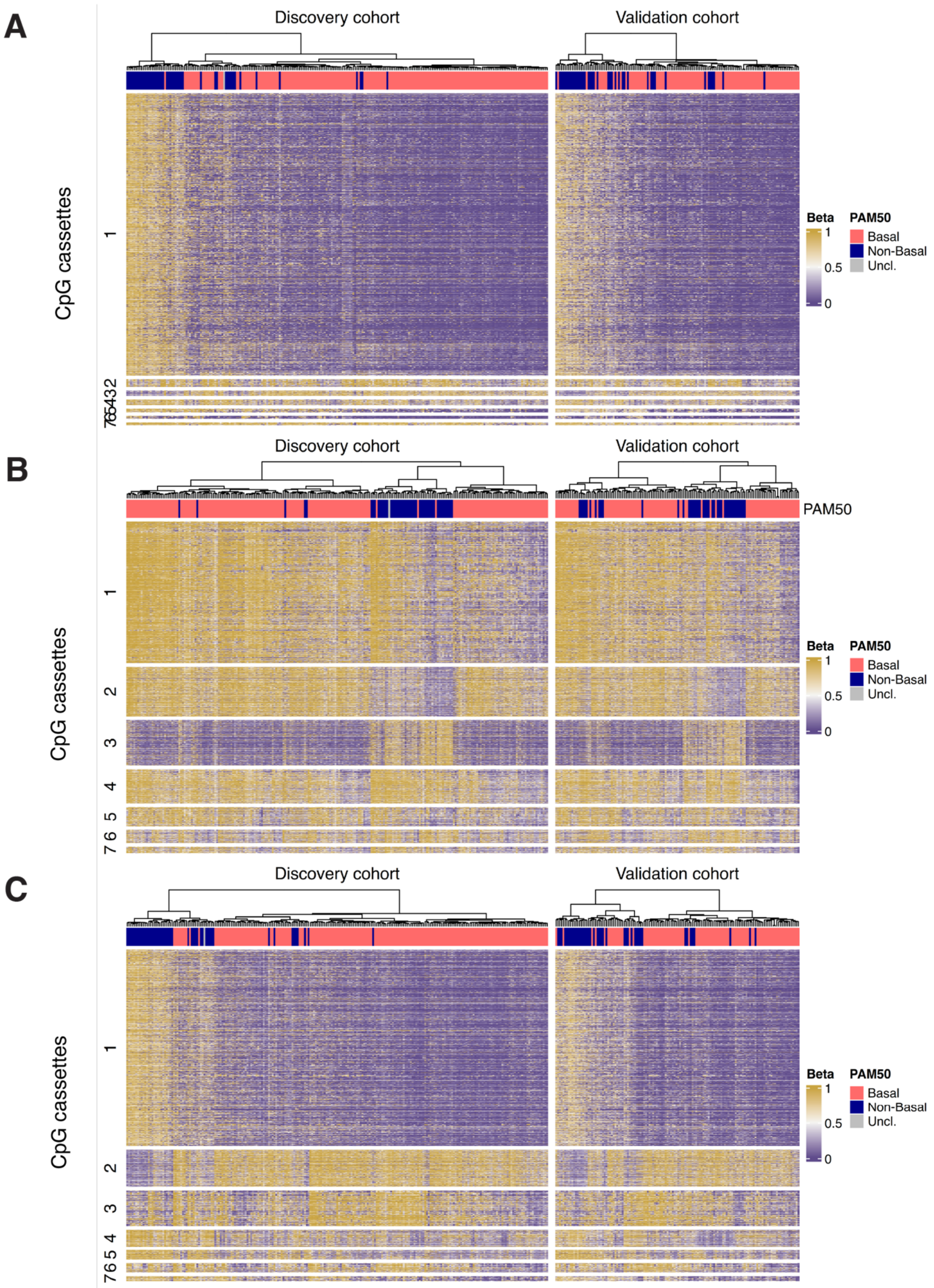

Supplementary Figure 3. CpG cassettes are detected consistently in the SCAN-B validation cohort. Heatmaps show the seven largest CpG context derived cassettes determined from the SCAN-B discovery and validation cohorts side by side: **A**) promoter CpGs, **B**) distal CpGs, and **C**) proximal CpGs. For each heatmap, the information included in the top annotations is detailed in respective heatmap's legends. Rows in the heatmaps correspond to CpGs, columns to samples. For each CpG context the patterns obtained from the discovery cohort are consistently preserved in the validation cohort.

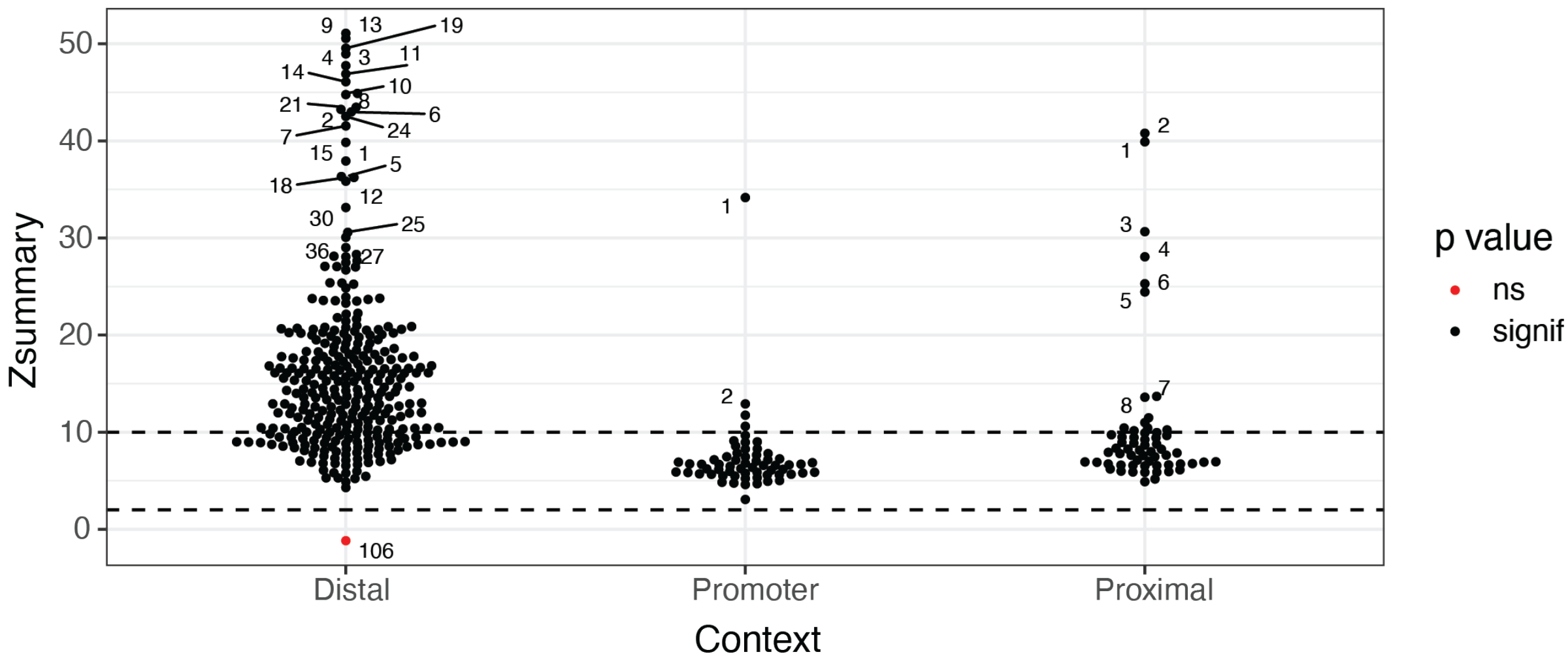

Supplementary Figure 4. Module preservation analysis of CpG context-specific cassettes in the SCAN-B validation cohort. Module preservation was evaluated using the modulePreservation() function from the WGCNA package. Cassettes with Zsummary>10 are considered highly preserved and Zsummary>2 moderately preserved, while a Zsummary<2 indicates no preservation. Preservation shows a high dependency on cassette size, with larger cassettes having higher Zsummary values. A single cassette, distal cassette 106, showed no preservation. Dot color represents permutation-based preservation significance from Bonferroni corrected p-values derived from 200 permutations.

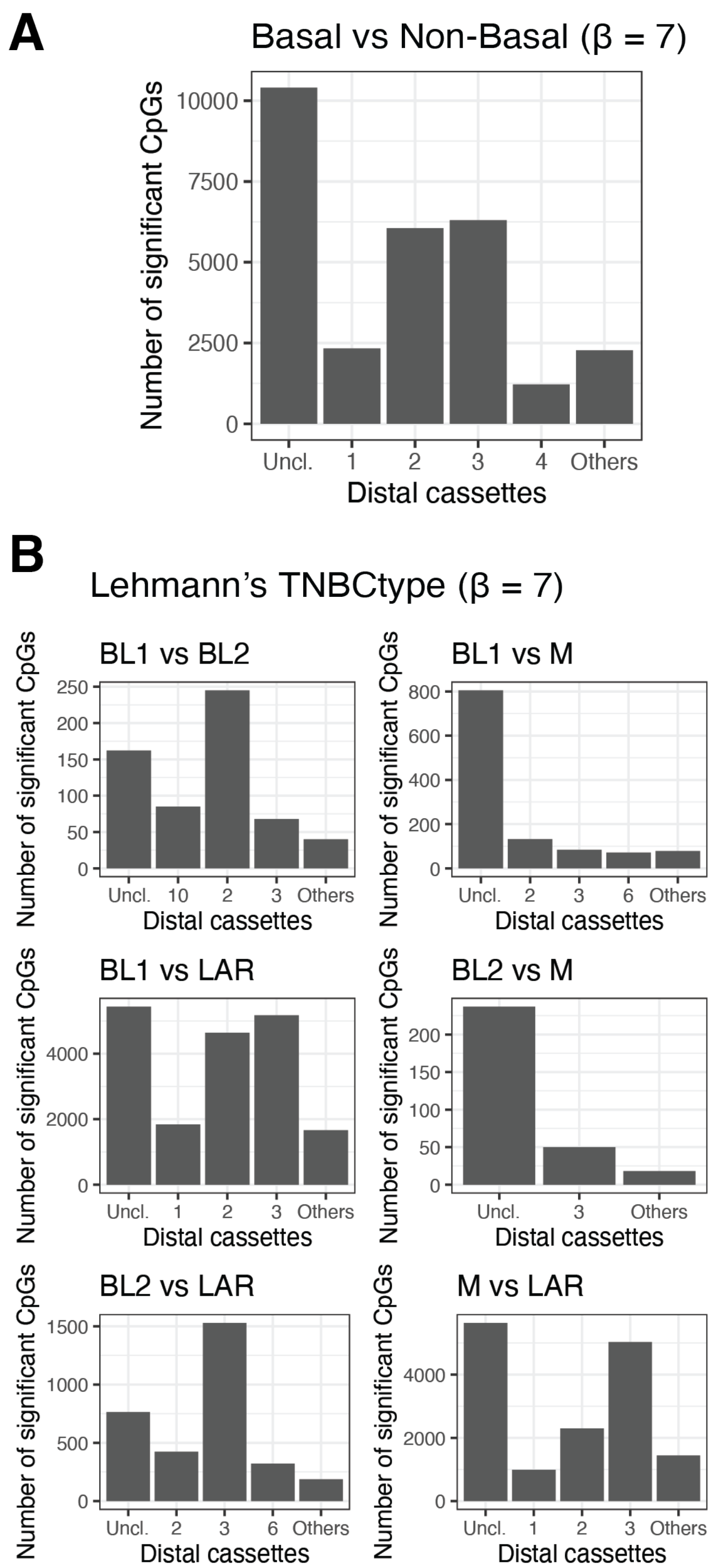

Supplementary Figure 5. Distal CpG cassette distribution of CpG sites selected by differential methylation analyses in the discovery cohort. The purity-adjusted beta values of all CpGs from the methylation cohort were analysed pairwise to detect differentially methylated CpG sites in Basal and non-Basal TNBC samples (**A**) and in every combination of the Lehmann et al. TNBC mRNA subtypes (TNBCtype) (**B**). The bar plots show the counts of CpGs selected (Bonferroni adjusted $p<0.05$) belonging to each of the distal cassettes. Cassettes with proportions of total CpGs below 0.05 were grouped in the "Others" category. Uncl. stands for unclustered CpGs.

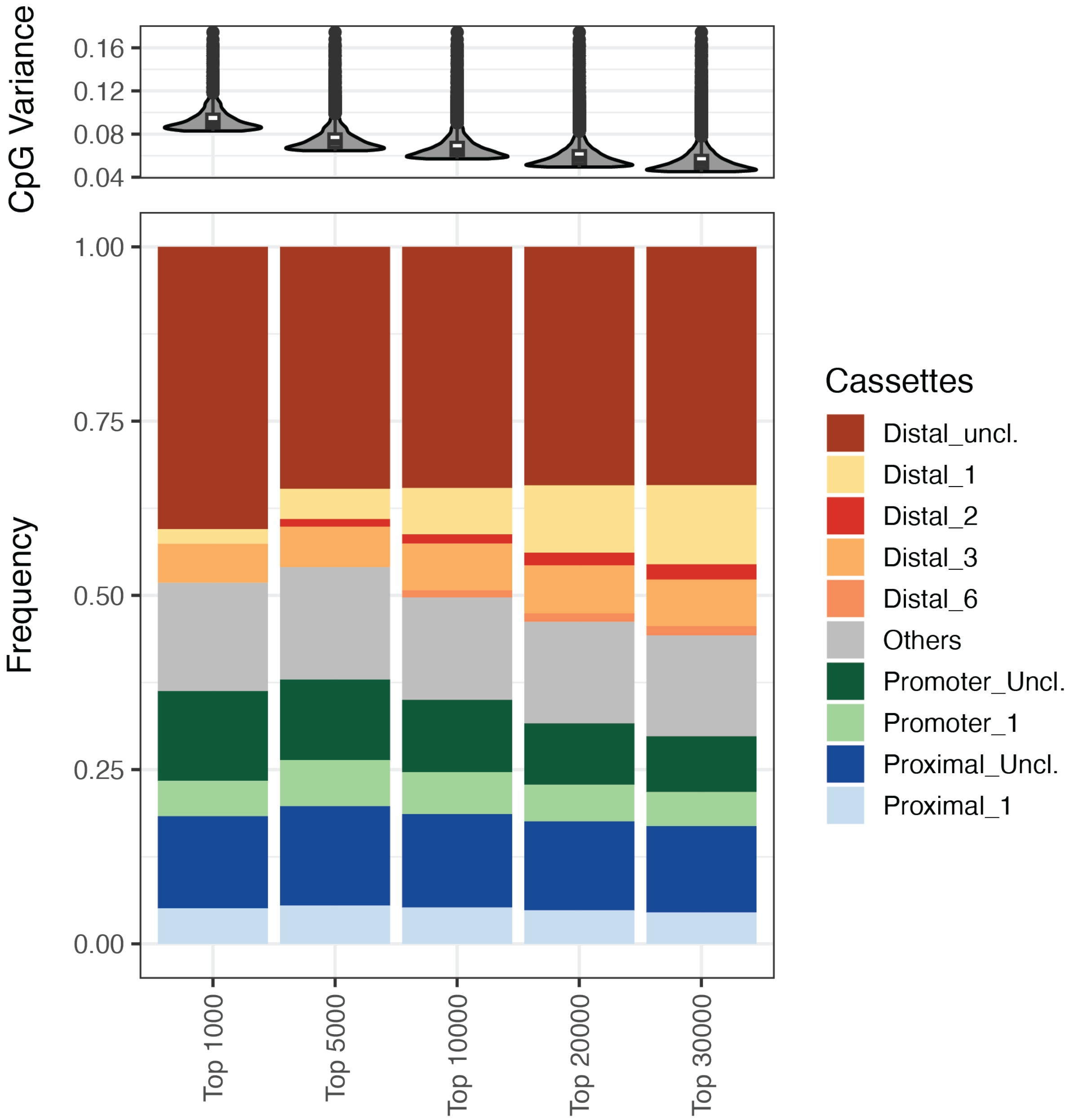

Supplementary Figure 6. Proportion of CpG cassettes per N top variable CpGs in the discovery cohort based on unadjusted methylation values. The variance used to select the CpGs was obtained from unadjusted beta values. The top violin plot shows the measured variance from unadjusted betas per group, and the bar plot the proportion of cassettes for respective N. CpGs below 1% of the total CpGs of each variance-based group were grouped and identified as "Others". Cassettes annotated as "Uncl." represent the unclassified CpGs.

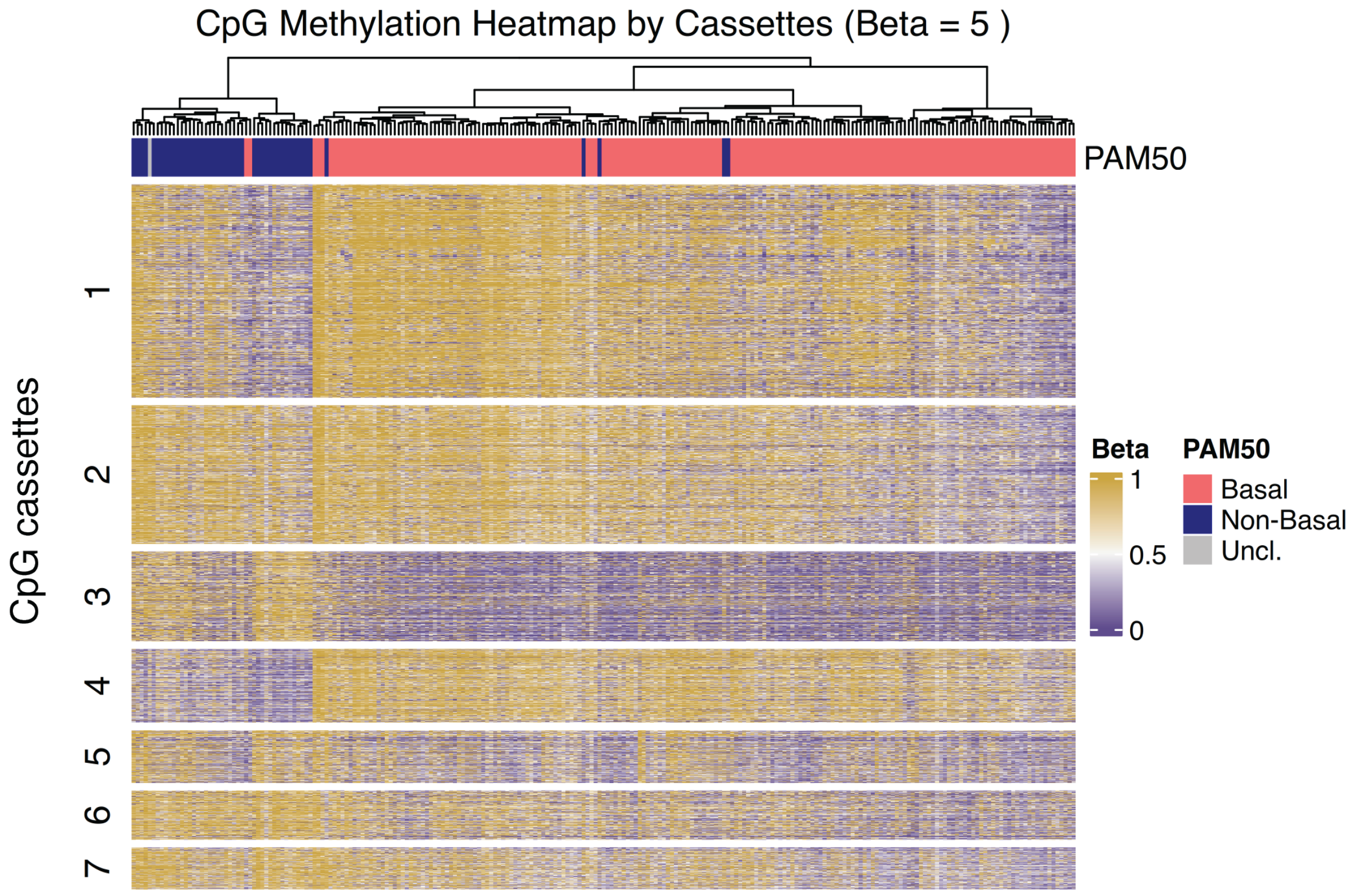

Supplementary Figure 7. Distal CpG cassettes detected in the discovery cohort using β=5 as the soft-thresholding value to minimize the proportion of unclustered CpGs. The remaining parameters and variance-threshold were kept unchanged from the ones described in the Methods section. Rows in the heatmap correspond to CpGs, columns to samples.

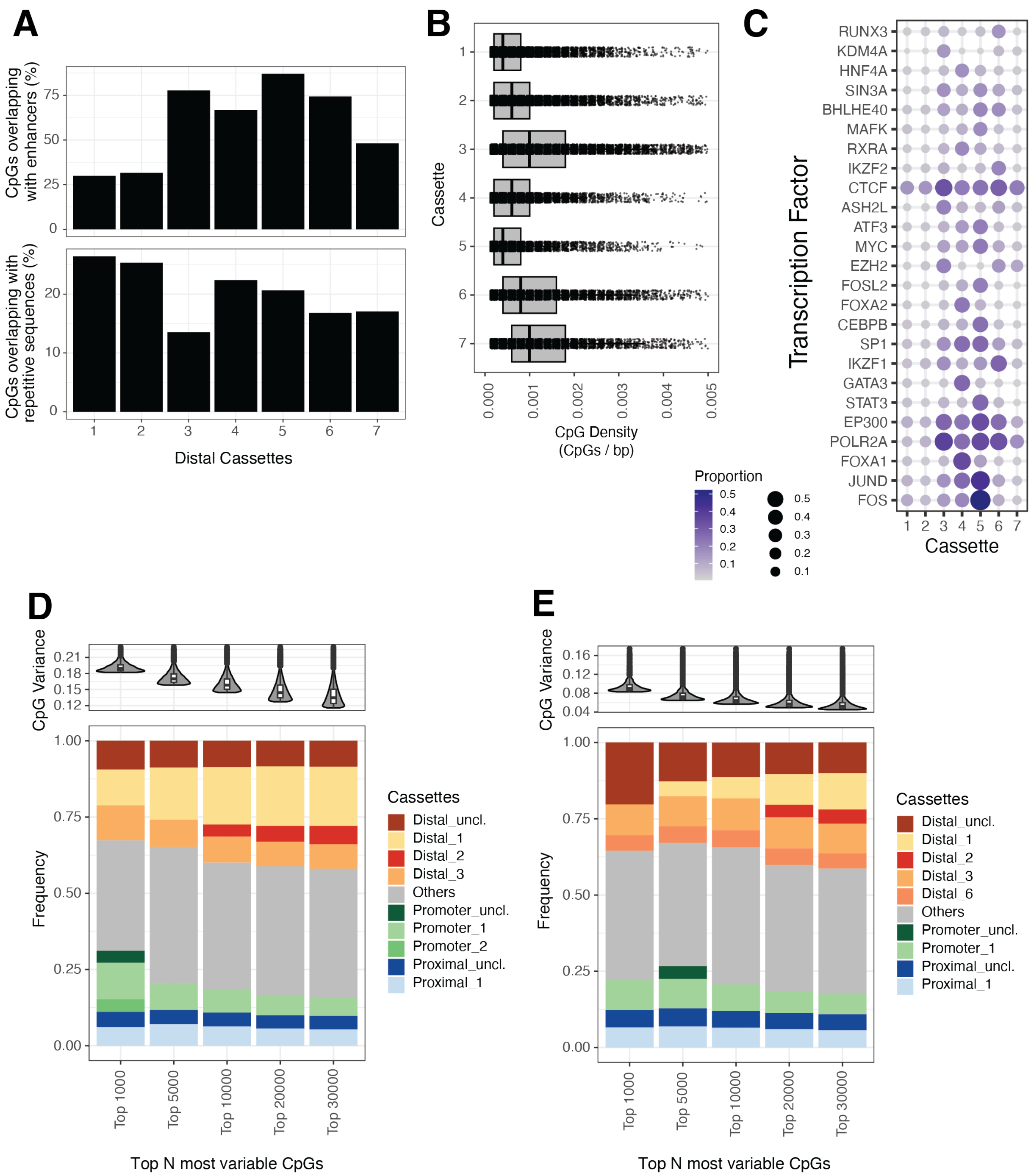

Supplementary Figure 8. Analysis of distal CpG cassettes and proportion of cassettes after variance-based filtering using β=5 as soft-thresholding power in the discovery cohort. A) Fraction of CpGs for the seven largest distal CpG cassettes overlapping with enhancers and repetitive regions. **B**) CpG density for the seven largest distal CpG cassettes. The density was determined using windows of 5000 base pairs. **C**) Fraction of CpGs in the seven largest distal cassettes overlapping with transcription factor binding sites. The transcription factors shown are the 25 ones with the highest pairwise differences between any two analyzed cassettes. **D**) Proportion of CpG cassettes per N top variable CpGs after variance-based filtering on purity-adjusted methylation data. The top violin plot shows the measured variance from adjusted beta values per group, and the bar plot the proportion of different CpG cassettes. CpGs below 1% of the total CpGs were grouped and identified as ”Others”. **E**) Proportion of CpG cassettes per N top variable CpGs after variance-based filtering on non-purity adjusted methylation data. The top violin plot shows the measured variance from unadjusted betas per group, and the bar plot the proportion of cassettes. CpGs below 1% of the total CpGs were grouped and identified as ”Others”. Cassettes annotated as “Uncl.” represent the unclassified CpGs.

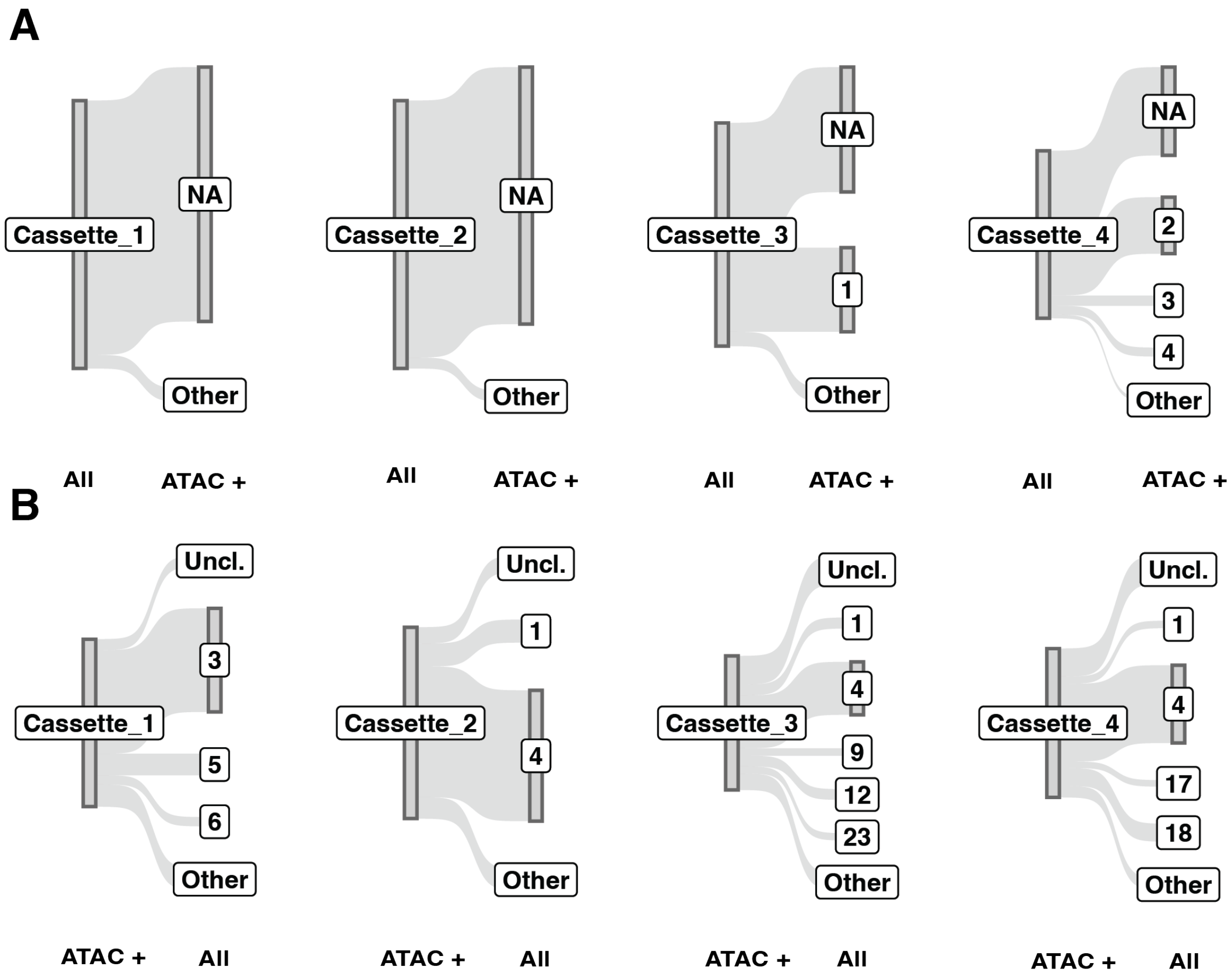

Supplementary Figure 9. Effect of ATAC-seq peak filtering on detected CpG cassettes when β=5 in the discovery cohort. A) Sankey plot between cassettes determined from all CpGs (left, first four) and from ATAC-seq peak filtered CpGs (right). The NA category represents filtered CpGs and "Other" includes cassettes whose proportion is below 4%. **B**) Sankey plot between CpG cassettes determined from ATAC-seq peak filtered CpGs (left, first four cassettes) and from all CpGs (right). The "Other" class includes cassettes whose proportion is below 4%.

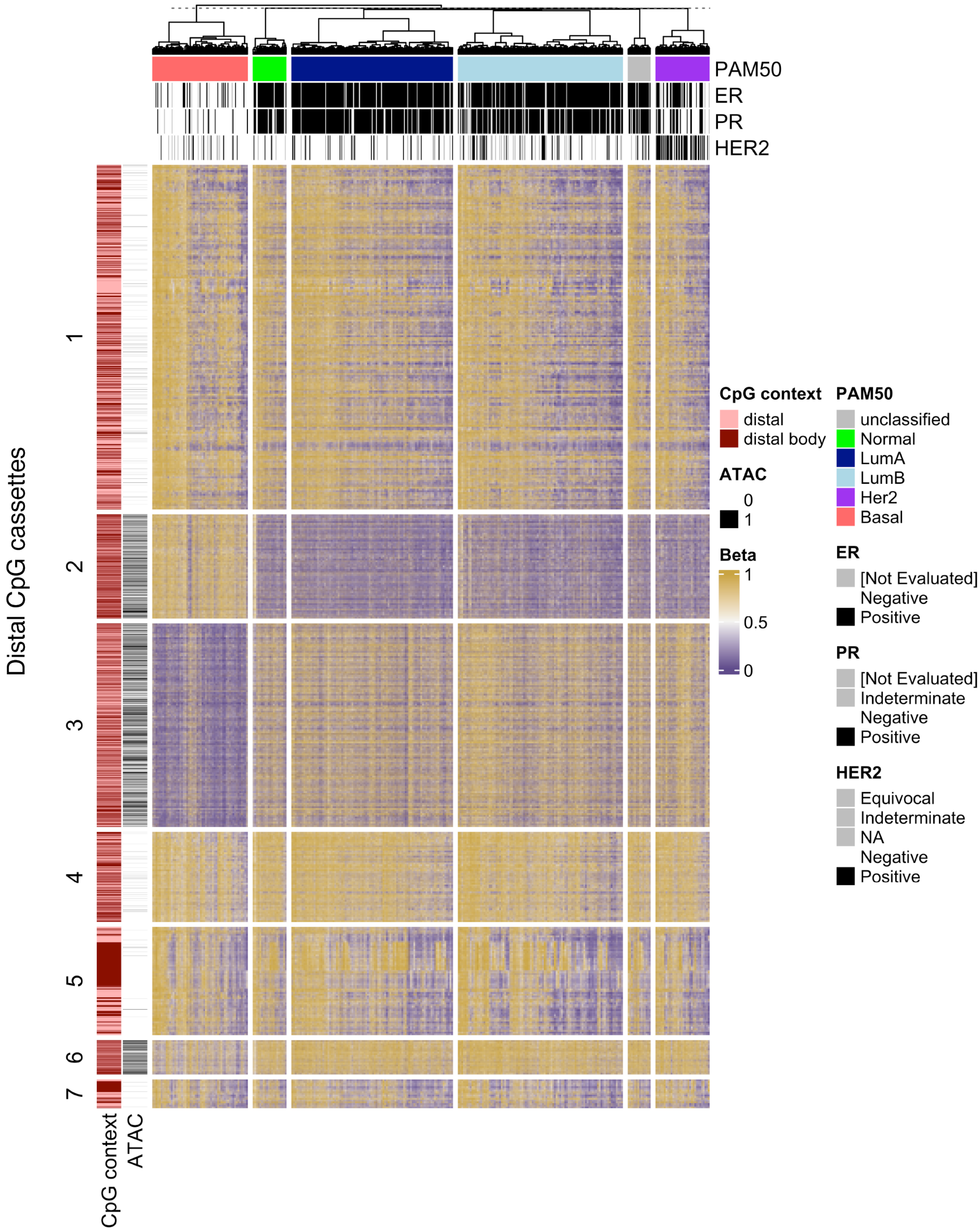

Supplementary Figure 10. Methylation patterns of distal CpG cassettes 1 to 7 in the TCGA breast cancer validation cohort. Heatmap of CpG beta values for CpGs in the seven largest distal cassettes across 645 breast cancers from the TCGA cohort. Only the CpGs overlapping between the Illumina EPIC and Infinium 450K methylation array platforms could be analyzed. The CpG cassette annotations displayed are taken from the annotations created for the EPIC dataset. The samples are ordered based on PAM50 subtypes. The information included in the top annotations is detailed in the heatmap's legends. Rows in the heatmap correspond to CpGs, columns to samples.

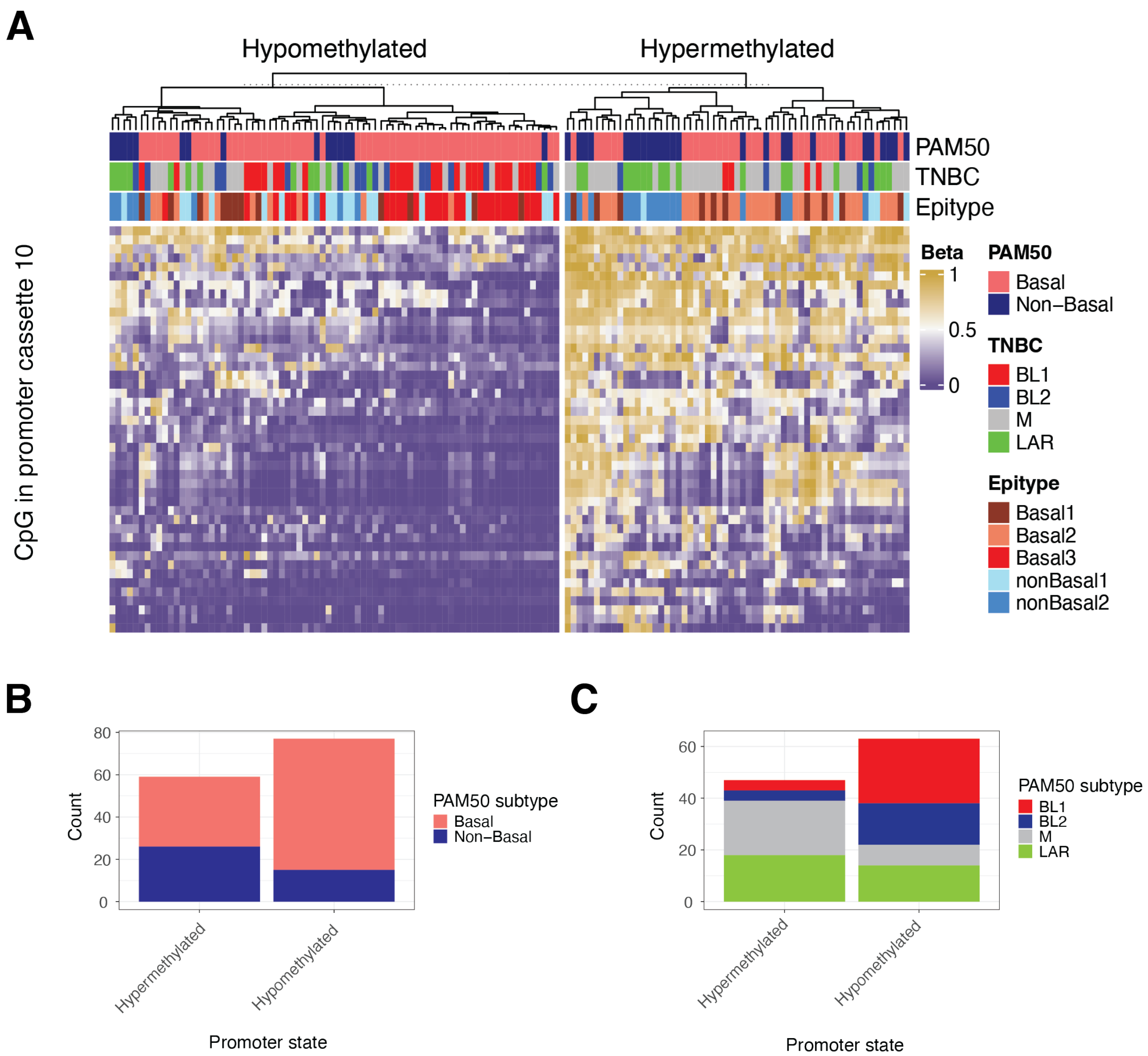

Supplementary Figure 11. Promoter CpG cassette 9 in the SCAN-B validation cohort. A) Methylation pattern of promoter cassette 9 in the SCAN-B validation cohort. The samples are grouped using k-means clustering with k=2 on the detected cassette's beta values in the validation cohort. The information included in the top annotations is detailed in the heatmap's legends. Rows in the heatmap correspond to CpGs, columns to samples. **B)** Distribution of PAM50 Basal/non-Basal subtypes per methylation state. **C)** Distribution of Lehmann et al. TNBC mRNA subtypes (TNBCtype) per methylation state.

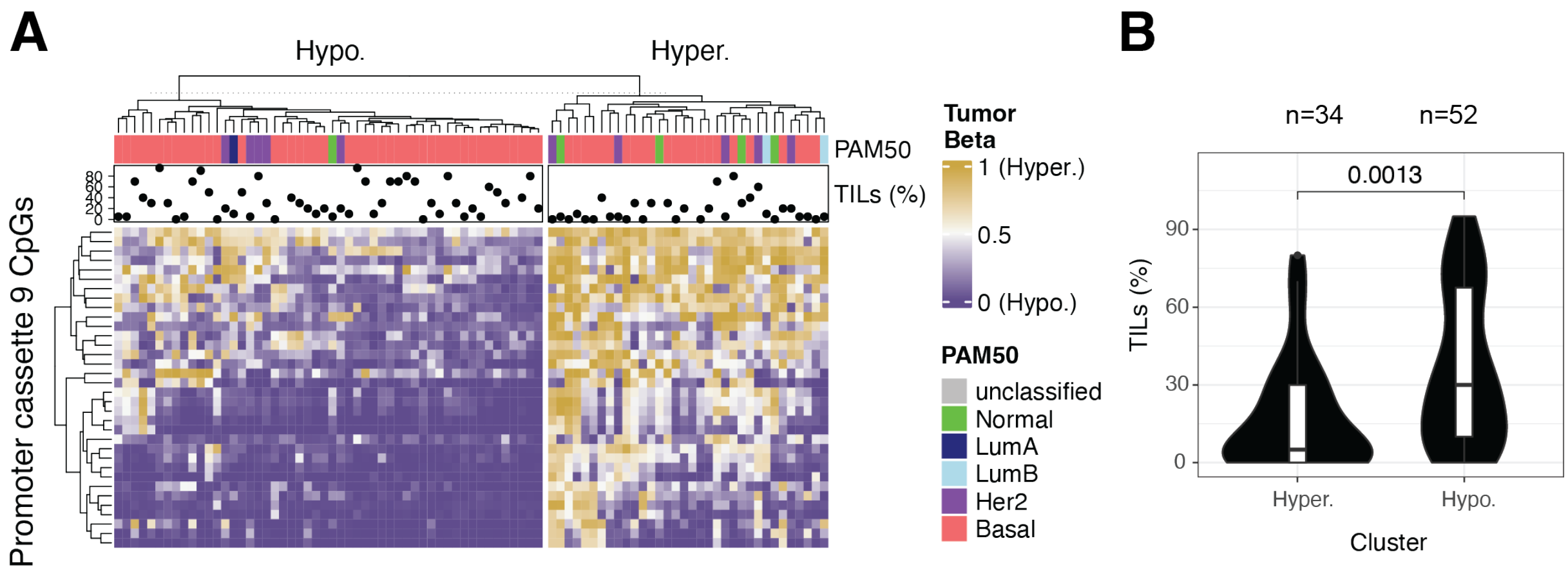

Supplementary Figure 12. Promoter cassette 9 in TNBC tumors from the TCGA breast cancer validation cohort. A) Methylation pattern of promoter cassette 9 in the external validation cohort. Only CpGs included in the available Illumina Infinium 450K methylation arrays in this cohort were used. The samples are grouped using k-means clustering with k=2 on the detected cassette's beta values in the external validation cohort. The information included in the top annotations is detailed in the heatmap's legends. Rows in the heatmap correspond to CpGs, columns to samples. **B)** Association between promoter cassette 9 and immune infiltration estimated by TILs in TCGA TNBCs. Samples were clustered as performed in A. Statistical significance was assessed using Wilcoxon's test. Hypo.: Hypomethylated. Hyper.: Hypermethylated.

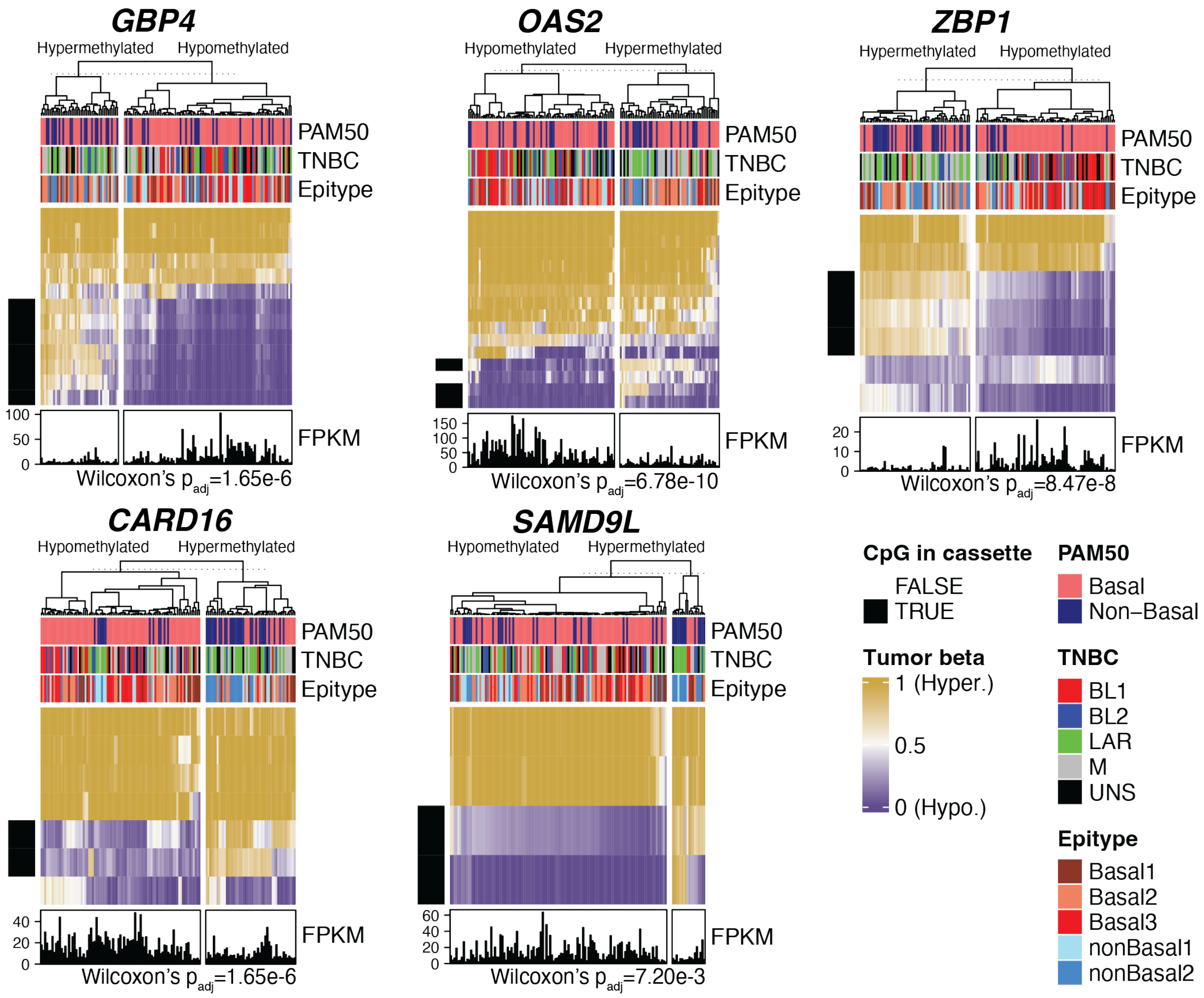

Supplementary Figure 13. Methylation state and gene expression of the five genes in promoter CpG cassette 9 in the SCAN-B validation cohort. Methylation state of CpGs overlapping the five genes targeted in promoter cassette 9 in the SCAN-B validation cohort. The samples are grouped using k-means clustering and k=2 on CpGs overlapping with the gene. The rows marked with black labels represent the CpGs included in promoter CpG cassette 9. FPKM gene expression of each gene per sample is shown in the bottom panel, which was significantly different per methylation cluster for the five genes analyzed using Wilcoxon's test and adjusted using the Benjamini-Hochberg method. The information included in the top annotations is detailed in the heatmap's legends. Rows in the heatmap correspond to CpGs, columns to samples.

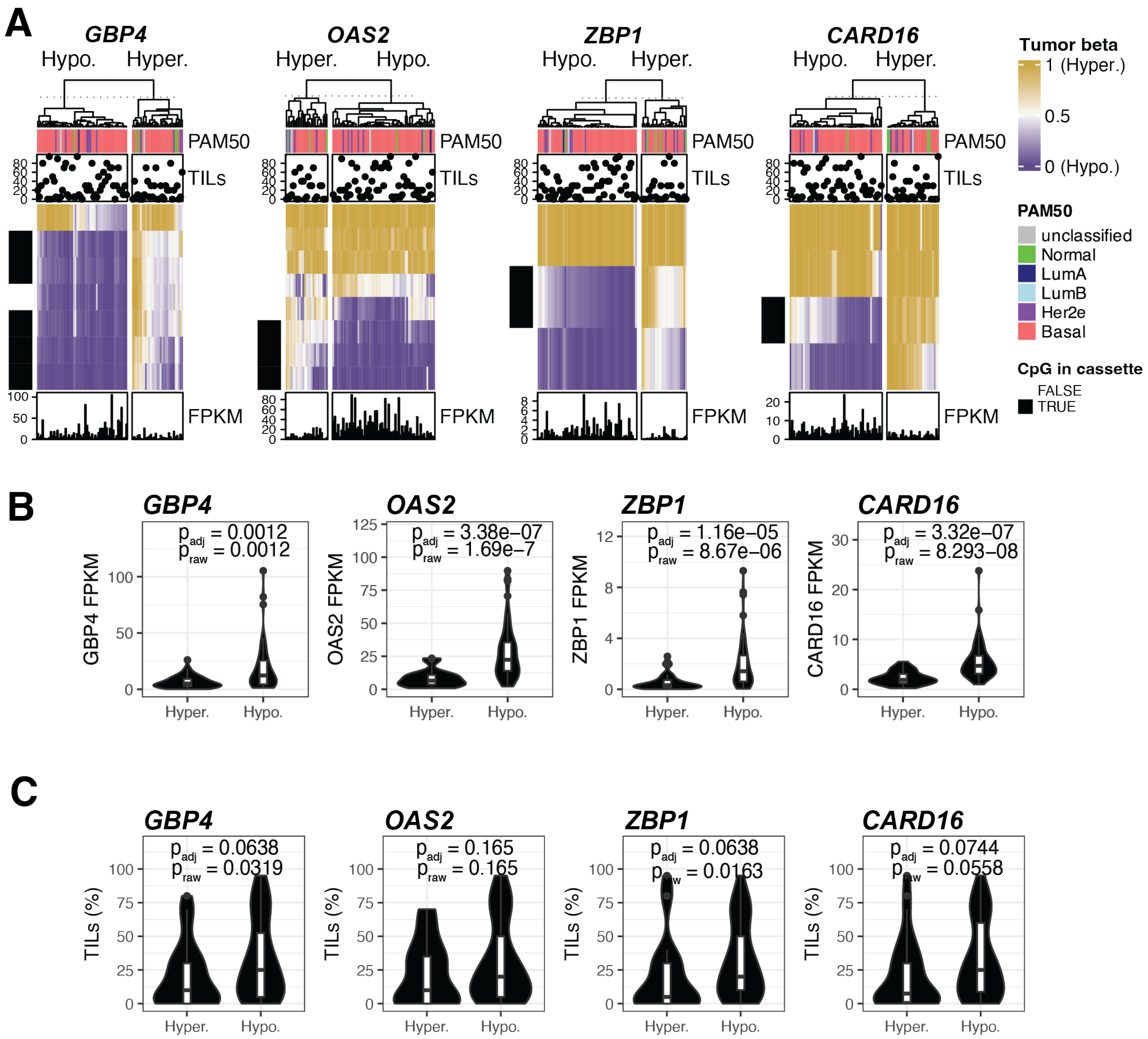

Supplementary Figure 14. Methylation state, gene expression, and association with TILs of the genes in promoter CpG cassette 9 in TNBCs from the TCGA breast cancer validation cohort. *SAMD9L* was excluded due to lack of CpG coverage in the available data. **A)** Methylation state of CpGs overlapping the four available genes targeted in promoter cassette 9 in the TCGA validation cohort. The samples are grouped using k-means clustering and k=2 on CpGs overlapping with the gene. The rows marked with black labels represent the CpGs included in promoter CpG cassette 9. The information included in the top annotations is detailed in the heatmap's legends. Rows in the heatmap correspond to CpGs, columns to samples. **B)** Association between methylation status defined in A with gene expression. Statistical significance was evaluated using Wilcoxon's test. **C)** Association between methylation status defined in A with TILs. Statistical significance was evaluated using Wilcoxon's test and adjusted using the Benjamini-Hochberg method.

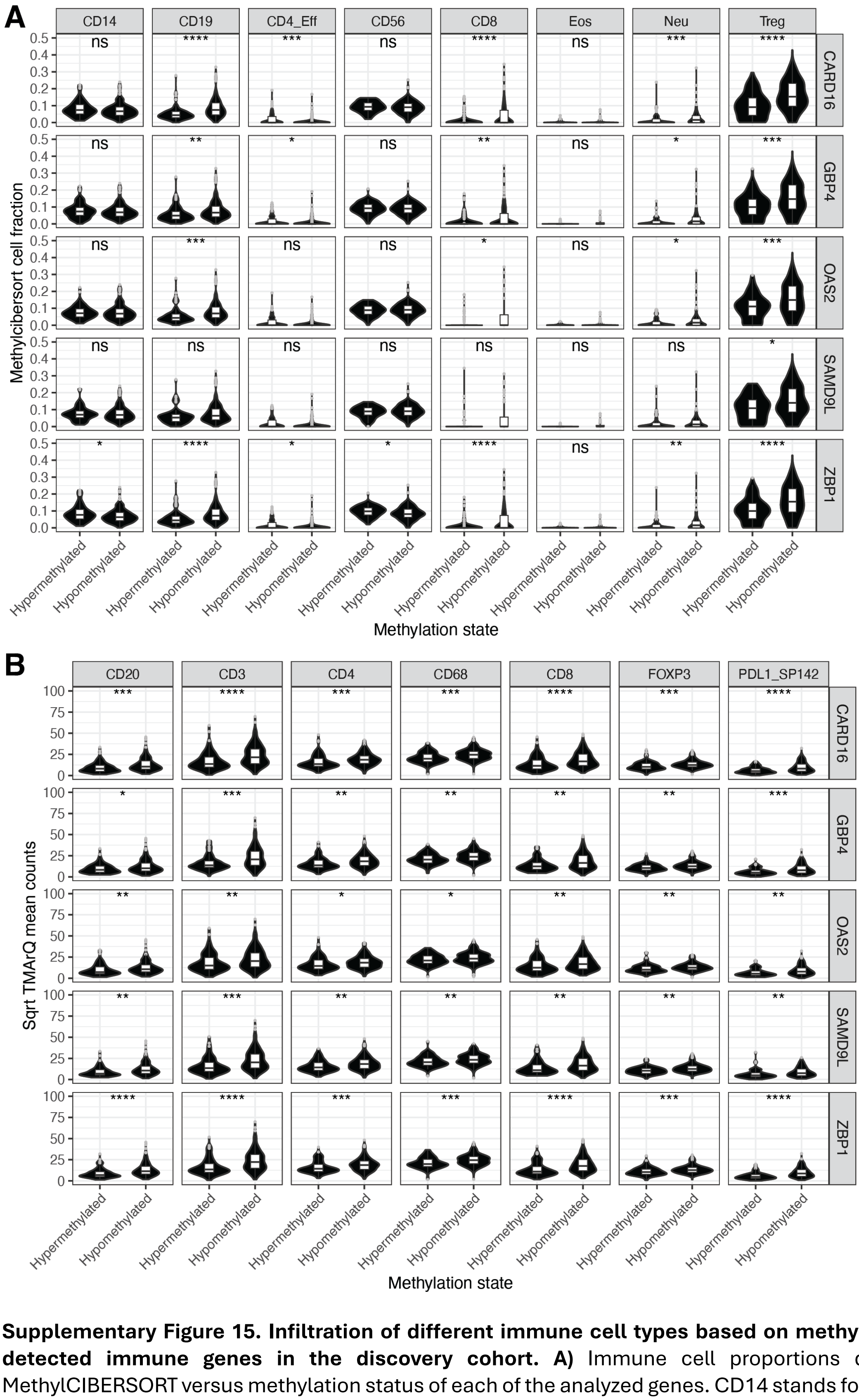

Supplementary Figure 15. Infiltration of different immune cell types based on methylation state of detected immune genes in the discovery cohort. A) Immune cell proportions determined by MethylCIBERSORT versus methylation status of each of the analyzed genes. CD14 stands for macrophages and monocytes, CD19 for B-cells, CD4_Eff stands for Helper T lymphocytes, CD56 for NK cells, CD8 for cytotoxic T lymphocytes, Neu for neutrophile and Treg for Regulatory T-cells. The methylation status was defined in Figure 5A. **B)** Square rooted TMArQ digital cell counts for immune cell type markers per methylation status of each of the analyzed genes. Here, PDL1 counts are based on IHC analysis using the SP142 antibody (see Roostee et al. for details). The statistical significance values displayed were obtained using Wilcoxon's test and adjusted per gene using the Benjamini-Hochberg method.

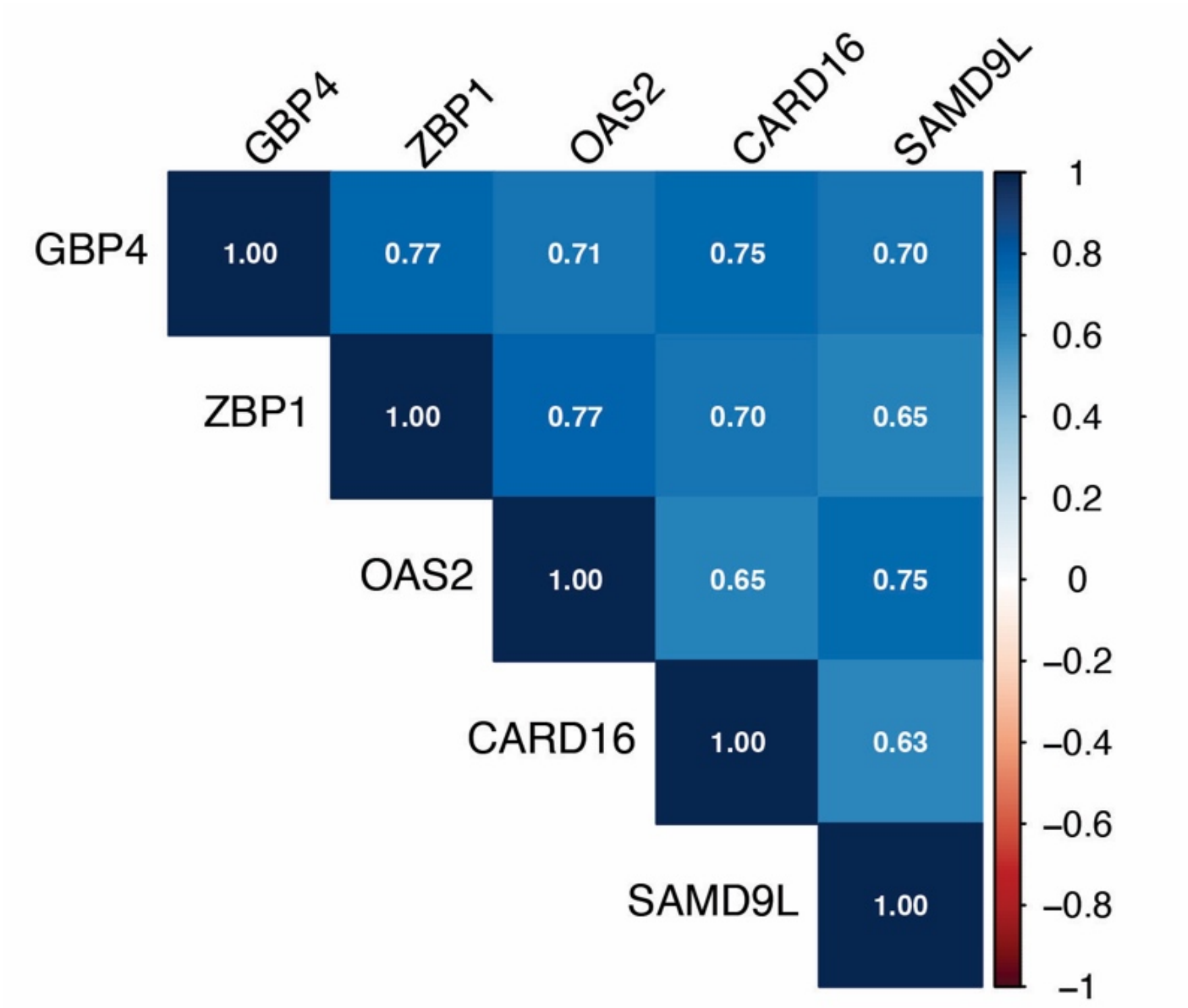

Supplementary Figure 16. Correlation plot between expression of the five genes from promoter CpG cassette 9 in the discovery cohort. The correlation values were calculated using the Spearman correlation coefficient from log1p-transformed FPKM gene expression values.

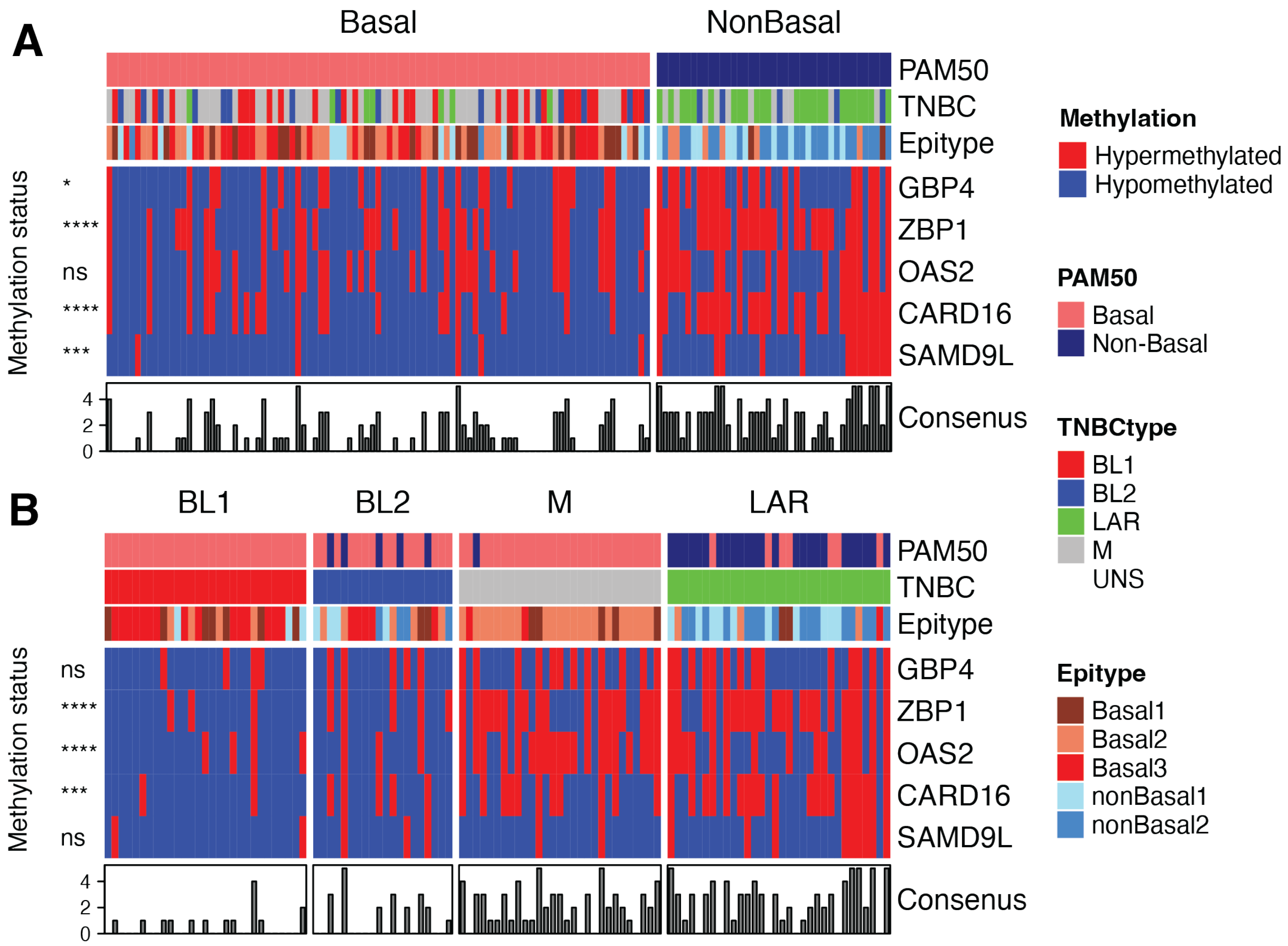

Supplementary Figure 17. Co-occurrence of the methylation patterns detected per gene in TNBC subtypes in the SCAN-B validation cohort. The samples are split based on Basal/non-Basal PAM50 groups (**A**) or Lehmann TNBCtype4 subtypes (**B**). The methylation status was defined in Supplementary Figure 12. Statistical significance values displayed on the left were obtained using the Chi-square test and adjusted using the Benjamini-Hochberg method. *: p<0.05, **: p<0.01, ****: p<0.0001. Columns in heatmaps represent samples, rows genes. The consensus bar plots display the number of hypermethylated genes per sample.

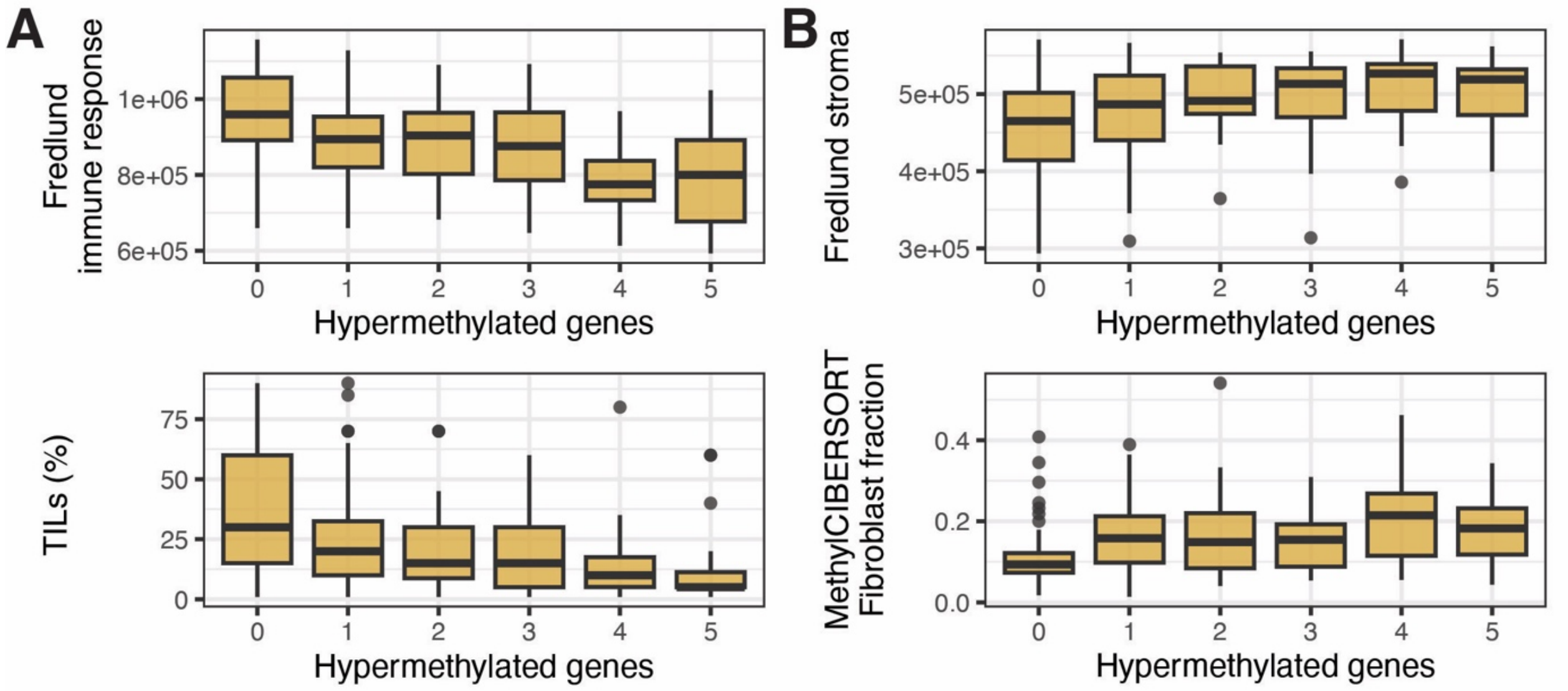

Supplementary Figure 18. Cumulative gene hypermethylation of cassette 9 genes versus tissue composition in the discovery cohort. A) Effect of cumulative hypermethylation of the five genes in promoter CpG cassette 9 versus immune infiltration measured by rank-based scores for a gene expression immune response metagene (top) and TILs (bottom). **B**) Effect of cumulative promoter hypermethylation in the five genes versus stromal estimates by a rank-based gene expression stroma metagene (top) and estimated fibroblast fraction by MethylCIBERSORT (bottom). The methylation status was defined in Figure 5A.

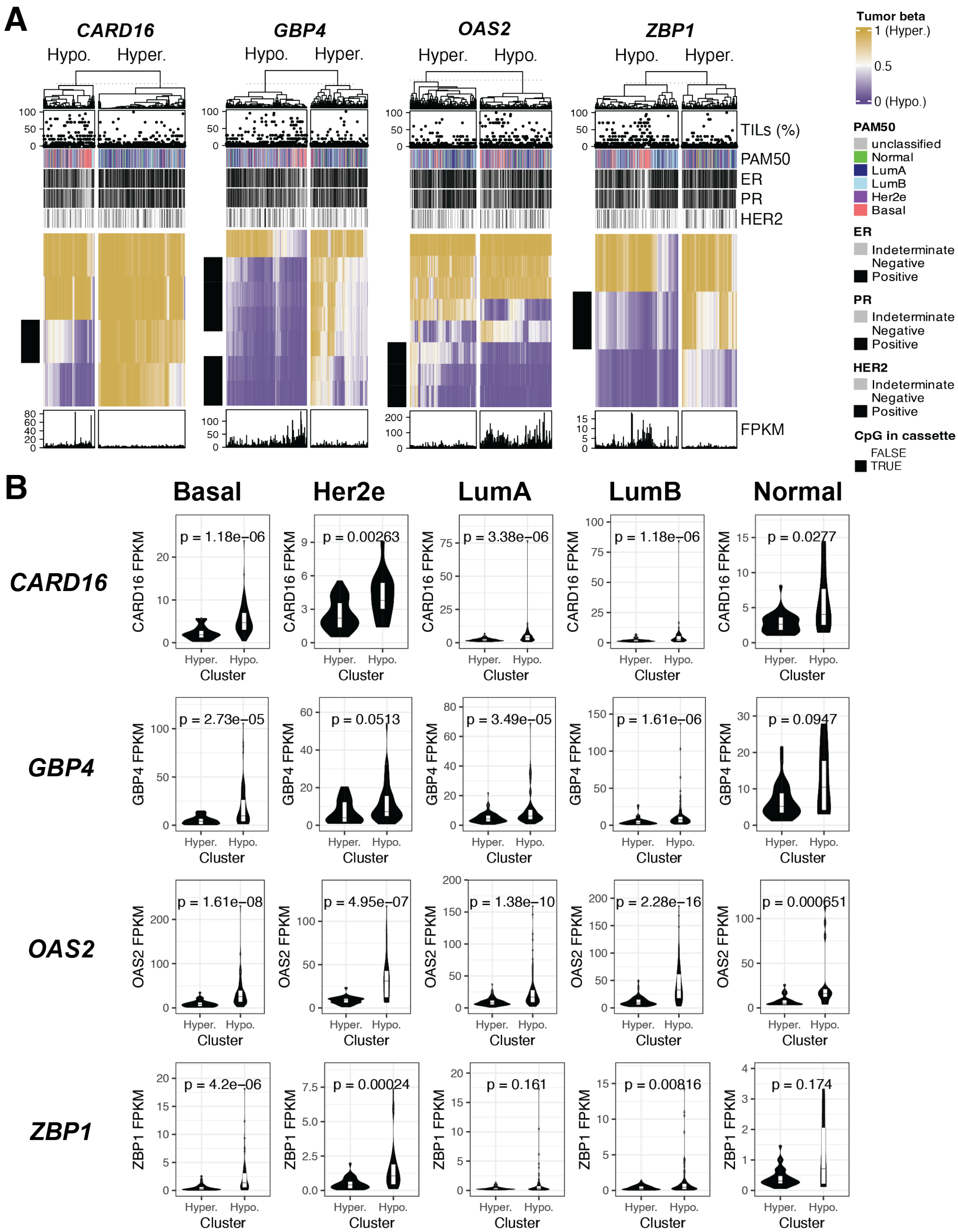

Supplementary Figure 19. Methylation state and gene expression of the five genes in promoter CpG cassette 9 in the TCGA breast cancer validation cohort. *SAMD9L* was excluded due to lack of CpG coverage in the available data. **A)** Methylation state of CpGs overlapping the four available genes targeted in promoter cassette 9 in the external validation cohort. The samples are grouped using k-means clustering and k=2 on CpGs overlapping with the gene. The rows marked with black labels represent the CpGs included in promoter CpG cassette 9. The information included in the top annotations is detailed in the heatmap's legends. Rows in the heatmap correspond to CpGs, columns to samples. Her2e: HER2-enriched. **B)** Association between methylation status defined in A with gene expression across PAM50 subtypes. Statistical significance was evaluated using Wilcoxon's test and adjusted per gene using the Benjamin-Hochberg method.

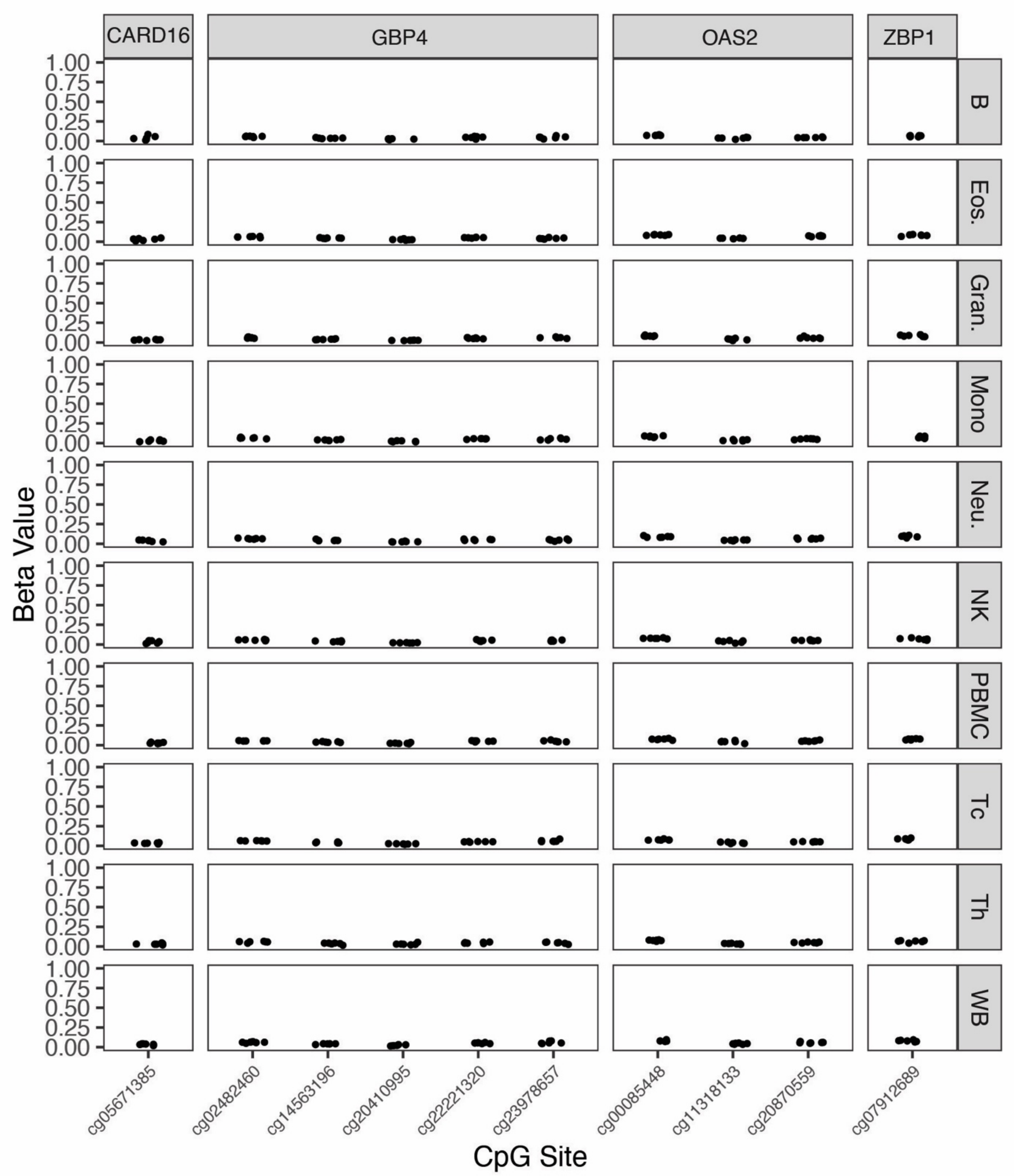

Supplementary Figure 20. Methylation state of CpGs in promoter CpG cassette 9 in immune cells from flow cytometry-sorted blood samples. CpGs not included were not available in the Reinius et al. dataset due to usage of the Illumina Infinium 450K platform. Cell types included are B-cells (B), eosinophiles (Eos.), granulocytes (Gran.), monocytes (Mono.), neutrophiles (Neu.), natural killer cells (NK cells), peripheral blood mononuclear cells (PBMC), cytotoxic T-cells (Tc), helper T-cells (Th), and whole blood (WB). Each cell type was profiled in six independent donors.

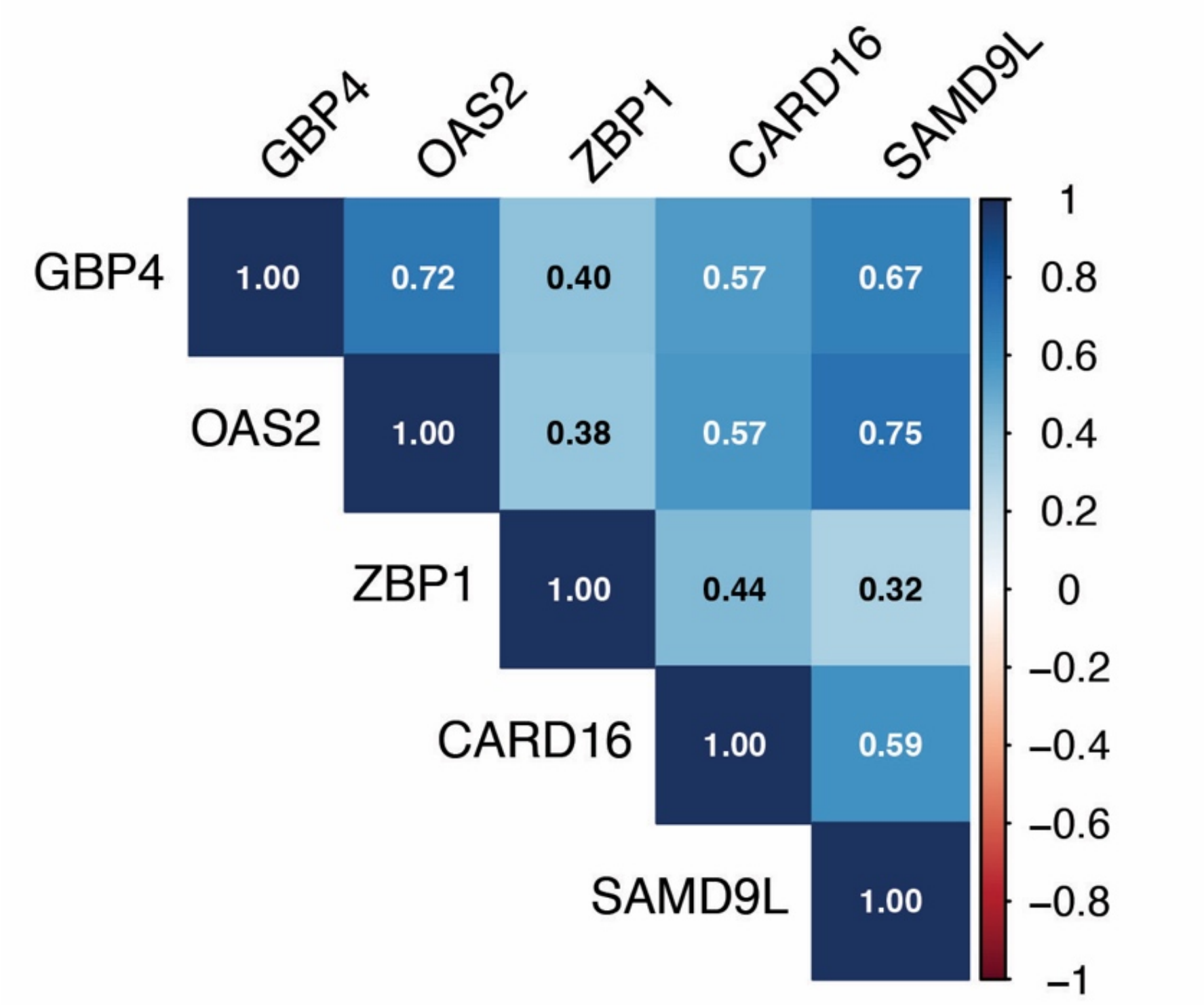

Supplementary Figure 21. Correlation plot between expression of the five genes from promoter CpG cassette 9 in bulk RNA-sequencing data from 34 TNBC cell lines. The correlation values were calculated using the Spearman correlation coefficient from log1p-transformed FPKM gene expression values.

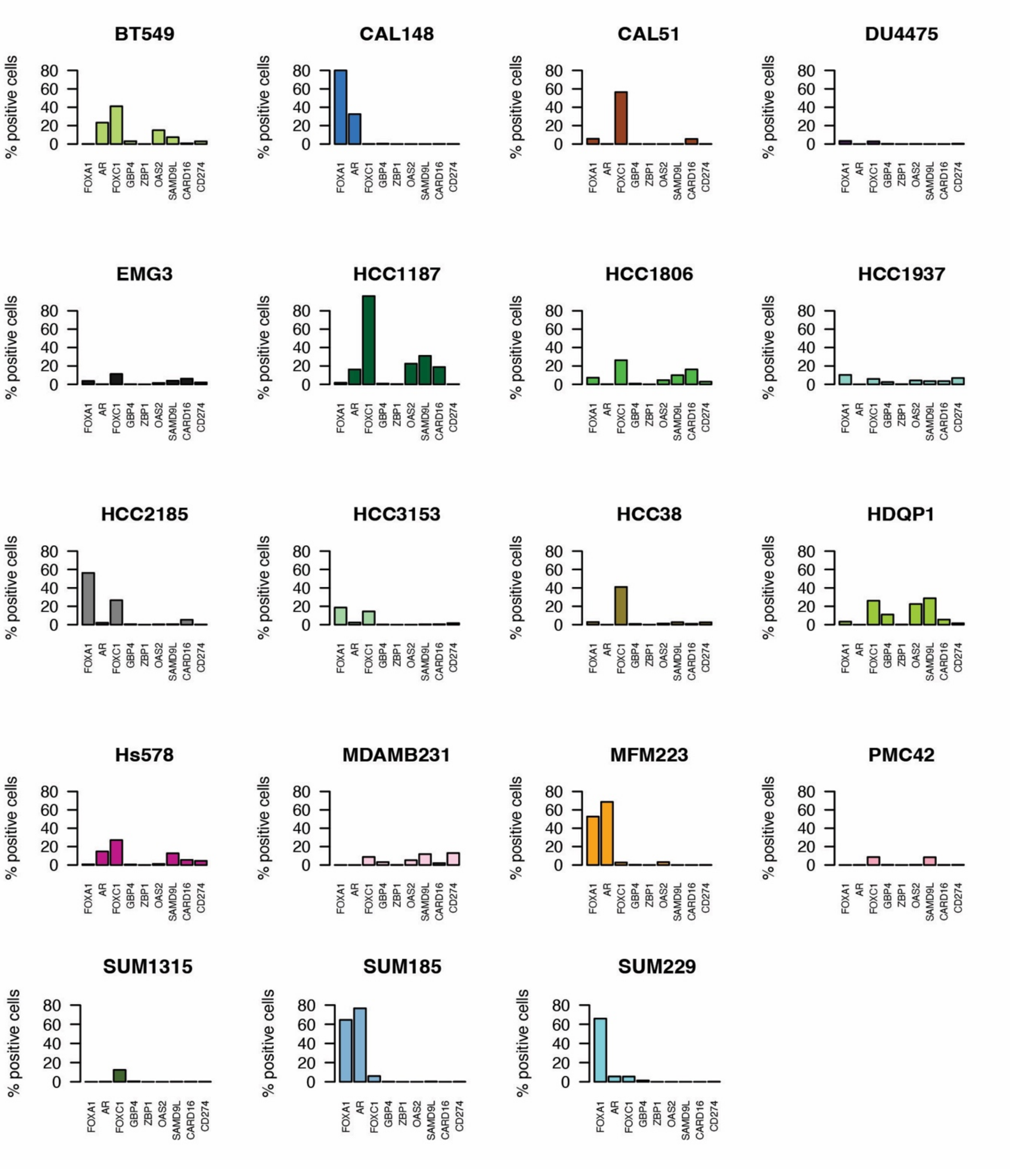

Supplementary Figure 22. scRNA-seq expression of the five genes in promoter CpG cassette 9 in 19 TNBC cell lines. The expression is summarized as proportion of cells with detected expression (>0 counts). Four additional genes were added to the plots as controls: for a Basal/non-Basal phenotype, *FOXA1*, *AR* (androgen receptor), and *FOXC1*; and for immune response, *CD274* (PD-L1).

Supplementary Table 1. SCAN-B TNBC dataset used for the spatial transcriptomics analyses.

	TMA cores	Unique samples
**N**	114	65
**Lymph node status**		
N0	73	42
N+	41	23
**Tumor grade**		
2	18	10
3	96	55
**PAM50 subtype**		
Basal-like	89	51
HER2-enriched	17	9
Luminal A	2	1
Luminal B	0	0
Normal-like	6	4
**TNBC subtype (Lehmann 4)**		
BL1	35	20
BL2	21	13
LAR	25	14
M	31	17
**Homologous Recombination status**		
Deficient	71	39
Proficient	43	26

Supplementary Table 2. Enriched GO terms in promoter cassette 1.

ONTOLOGY	TERM	N	DE	P.DE	FDR
BP	neurogenesis	629	292	3.59E-12	5.85E-08
BP	system development	1456	592	1.40E-11	1.14E-07
BP	multicellular organism development	1636	655	3.39E-11	1.38E-07
BP	nervous system development	944	408	2.64E-11	1.38E-07
BP	plasma membrane bounded cell projection organization	514	239	5.28E-11	1.72E-07
BP	generation of neurons	555	257	1.28E-10	3.48E-07
BP	cell projection organization	527	242	1.80E-10	4.20E-07
BP	neuron differentiation	537	249	2.60E-10	5.30E-07
BP	anatomical structure morphogenesis	1009	415	7.12E-09	1.16E-05
BP	neuron projection development	374	177	6.97E-09	1.16E-05
BP	enzyme-linked receptor protein signaling pathway	348	160	1.84E-08	2.73E-05
BP	anatomical structure development	2063	779	5.28E-08	7.17E-05
BP	neuron development	438	199	5.85E-08	7.34E-05
BP	axon development	212	107	2.17E-07	2.53E-04
BP	central nervous system development	394	179	4.26E-07	4.63E-04
BP	negative regulation of Wnt signaling pathway	64	41	8.65E-07	8.82E-04
BP	axonogenesis	188	95	1.00E-06	9.64E-04
BP	regulation of signaling	1120	435	1.14E-06	0.001037
BP	regulation of cell communication	1126	437	1.21E-06	0.0010416
BP	developmental process	2215	822	1.47E-06	0.00119605
BP	cell morphogenesis involved in neuron differentiation	232	111	2.73E-06	0.00202276
BP	plasma membrane bounded cell projection morphogenesis	262	123	2.64E-06	0.00202276
CC	cell junction	815	328	3.44E-06	0.00233995
BP	cell projection morphogenesis	263	123	3.32E-06	0.00233995
BP	sensory organ development	250	119	3.74E-06	0.00241176
BP	regulation of anatomical structure morphogenesis	305	138	3.84E-06	0.00241176
BP	cell differentiation	1532	585	4.82E-06	0.00262773
CC	synapse	586	246	4.83E-06	0.00262773
BP	cellular developmental process	1532	585	4.82E-06	0.00262773
BP	renal tubule development	37	27	4.75E-06	0.00262773
BP	regulation of developmental process	811	328	5.00E-06	0.00263024
BP	cellular response to endogenous stimulus	407	175	5.16E-06	0.00263065
BP	Wnt signaling pathway	130	69	5.45E-06	0.00269679
BP	neuron projection morphogenesis	257	120	5.94E-06	0.00284903
BP	animal organ development	1076	421	7.81E-06	0.00364227
BP	kidney epithelium development	56	36	9.37E-06	0.00424711
BP	tube development	402	175	1.02E-05	0.00447838
BP	regulation of neuron projection development	151	76	1.17E-05	0.00503902
BP	cell morphogenesis	361	159	1.29E-05	0.0053829
BP	animal organ morphogenesis	400	176	1.83E-05	0.00746458
BP	cellular response to growth factor stimulus	242	110	1.96E-05	0.00780842
BP	regulation of nervous system development	159	80	2.07E-05	0.00802482
BP	nephron tubule development	35	25	2.20E-05	0.00833282
BP	response to endogenous stimulus	496	206	2.25E-05	0.00833699
BP	regulation of neurogenesis	124	65	2.42E-05	0.00878592
BP	central nervous system neuron differentiation	113	61	2.56E-05	0.00908263
BP	nephron morphogenesis	28	21	2.86E-05	0.00991706
BP	renal tubule morphogenesis	28	21	3.49E-05	0.01168066
BP	response to growth factor	250	112	3.51E-05	0.01168066
BP	nephron epithelium morphogenesis	27	20	6.95E-05	0.0224537
BP	negative regulation of canonical Wnt signaling pathway	48	30	7.02E-05	0.0224537
BP	nephron epithelium development	45	29	7.81E-05	0.02450112
BP	regulation of system process	245	112	8.44E-05	0.02550465
BP	regulation of neuron differentiation	80	45	8.37E-05	0.02550465
BP	regulation of Wnt signaling pathway	108	56	9.27E-05	0.02700571
BP	head development	295	132	9.18E-05	0.02700571
CC	presynapse	249	113	9.56E-05	0.02737288
CC	basement membrane	45	28	1.13E-04	0.03136251
BP	tube morphogenesis	326	141	1.13E-04	0.03136251
BP	skeletal system development	205	96	1.16E-04	0.03153715
BP	regulation of signal transduction	941	358	1.18E-04	0.03167852
CC	extracellular matrix	263	115	1.22E-04	0.03203521
BP	cell surface receptor protein tyrosine kinase signaling pathway	226	99	1.45E-04	0.03703964

BP	regulation of trans-synaptic signaling	209	97	1.45E-04	0.03703964
CC	external encapsulating structure	264	115	1.53E-04	0.03790838
BP	renal system development	113	58	1.53E-04	0.03790838
BP	nephron tubule morphogenesis	26	19	1.69E-04	0.04109599
BP	regulation of plasma membrane bounded cell projection organization	194	89	1.84E-04	0.04405355
BP	embryonic morphogenesis	236	108	1.92E-04	0.0443221
BP	modulation of chemical synaptic transmission	208	96	1.94E-04	0.0443221
BP	canonical Wnt signaling pathway	89	47	1.96E-04	0.0443221
CC	glutamatergic synapse	187	86	1.95E-04	0.0443221
BP	ureteric bud development	37	24	2.06E-04	0.04491834
BP	mesonephric epithelium development	37	24	2.06E-04	0.04491834
BP	mesonephric tubule development	37	24	2.06E-04	0.04491834
CC	axon	252	112	2.15E-04	0.04619089
BP	muscle system process	205	93	2.19E-04	0.04644583
BP	telencephalon development	113	57	2.27E-04	0.04741132

Supplementary Table 3. Enriched GO terms in proximal cassette 1.

ONTOLOGY	TERM	N	DE	P.DE	FDR
BP	generation of neurons	1059	368	5.36E-16	1.06E-11
BP	nervous system development	1755	547	2.32E-15	2.30E-11
BP	neuron differentiation	1007	350	4.04E-15	2.66E-11
BP	system development	2678	761	1.18E-14	5.84E-11
BP	neurogenesis	1219	402	1.78E-14	7.05E-11
BP	multicellular organism development	3047	819	1.32E-11	4.36E-08
BP	neuron projection development	703	241	2.16E-11	6.09E-08
BP	plasma membrane bounded cell projection organization	1007	308	5.63E-11	1.39E-07
BP	cell projection organization	1028	311	1.53E-10	3.36E-07
BP	regulation of nervous system development	323	128	2.33E-10	4.60E-07
BP	cell morphogenesis involved in neuron differentiation	423	158	2.65E-10	4.77E-07
BP	neuron development	811	269	3.67E-10	6.03E-07
BP	axon development	374	144	4.89E-10	7.43E-07
BP	animal organ development	1996	570	5.71E-10	8.06E-07
BP	anatomical structure morphogenesis	1813	525	7.08E-10	9.33E-07
BP	axonogenesis	332	131	8.91E-10	1.10E-06
BP	regulation of neuron differentiation	139	74	9.55E-10	1.11E-06
CC	synapse	1080	330	1.17E-09	1.29E-06
BP	regulation of developmental process	1571	449	1.30E-09	1.35E-06
BP	animal organ morphogenesis	718	248	3.66E-09	3.41E-06
BP	cell projection morphogenesis	478	170	3.50E-09	3.41E-06
BP	plasma membrane bounded cell projection morphogenesis	475	169	3.79E-09	3.41E-06
BP	neuron projection morphogenesis	462	164	9.42E-09	8.09E-06
BP	central nervous system development	713	246	1.29E-08	1.06E-05
BP	brain development	503	185	1.80E-08	1.42E-05
BP	developmental process	4063	1006	2.18E-08	1.60E-05
BP	positive regulation of nervous system development	197	85	2.14E-08	1.60E-05
BP	forebrain development	289	122	2.55E-08	1.80E-05
BP	positive regulation of developmental process	865	269	2.74E-08	1.87E-05
BP	regulation of plasma membrane bounded cell projection organization	410	141	3.03E-08	2.00E-05
BP	sensory organ development	448	162	3.85E-08	2.45E-05
BP	cell-cell signaling	915	288	4.07E-08	2.51E-05
BP	anatomical structure development	3771	946	4.25E-08	2.55E-05
BP	head development	538	193	4.71E-08	2.73E-05
BP	central nervous system neuron differentiation	188	88	5.31E-08	3.00E-05
CC	neuron projection	864	261	5.55E-08	3.05E-05
BP	regulation of cell differentiation	1026	306	6.13E-08	3.27E-05
BP	cell morphogenesis	669	213	6.68E-08	3.47E-05
BP	regulation of cell projection organization	421	142	7.61E-08	3.86E-05
BP	tube development	763	245	9.27E-08	4.58E-05
BP	regulation of neuron projection development	304	112	1.18E-07	5.70E-05
BP	cell differentiation	2800	724	1.25E-07	5.75E-05

BP	cellular developmental process	2800	724	1.25E-07	5.75E-05
BP	regulation of neurogenesis	258	101	1.28E-07	5.75E-05
BP	embryonic morphogenesis	437	163	1.49E-07	6.52E-05
CC	cell junction	1510	413	1.69E-07	7.27E-05
BP	kidney epithelium development	109	56	1.75E-07	7.34E-05
BP	tissue development	1338	388	1.95E-07	8.04E-05
BP	regulation of multicellular organismal development	938	278	2.86E-07	1.15E-04
BP	forebrain regionalization	23	20	5.80E-07	2.29E-04
BP	embryonic organ morphogenesis	231	98	5.93E-07	2.30E-04
BP	embryonic organ development	338	129	6.05E-07	2.30E-04
BP	telencephalon development	202	85	6.75E-07	2.52E-04
BP	ureteric bud development	75	42	9.18E-07	3.18E-04
BP	nephron epithelium development	87	46	8.82E-07	3.18E-04
BP	mesonephric epithelium development	75	42	9.18E-07	3.18E-04
BP	mesonephric tubule development	75	42	9.18E-07	3.18E-04
BP	branching involved in ureteric bud morphogenesis	50	31	1.28E-06	4.36E-04
BP	axon guidance	182	77	1.57E-06	5.16E-04
BP	neuron projection guidance	182	77	1.57E-06	5.16E-04
CC	somatodendritic compartment	564	180	2.11E-06	6.84E-04
BP	regulation of system process	407	133	2.21E-06	7.03E-04
BP	mesonephric tubule morphogenesis	53	32	2.55E-06	8.00E-04
CC	dendrite	414	138	2.69E-06	8.31E-04
BP	renal system development	220	87	2.90E-06	8.82E-04
BP	tissue morphogenesis	427	151	3.04E-06	9.11E-04
BP	nephron tubule morphogenesis	58	34	3.14E-06	9.25E-04
BP	nephron tubule development	73	40	3.26E-06	9.46E-04
BP	metanephros development	67	39	3.59E-06	0.00102493
CC	dendritic tree	416	138	3.63E-06	0.00102493
BP	nephron morphogenesis	61	35	3.86E-06	0.00107543
BP	epithelial tube morphogenesis	225	90	3.93E-06	0.00107727
BP	nephron development	113	52	4.14E-06	0.00111919
BP	kidney development	209	83	4.39E-06	0.0011715
BP	ureteric bud morphogenesis	52	31	4.51E-06	0.00118828
BP	nephron epithelium morphogenesis	59	34	4.71E-06	0.00122497
BP	mesonephros development	78	42	5.23E-06	0.00132579
BP	negative regulation of developmental process	631	196	5.21E-06	0.00132579
BP	renal tubule morphogenesis	62	35	5.31E-06	0.00132858
BP	renal tubule development	78	41	5.48E-06	0.00135324
BP	kidney morphogenesis	73	39	5.79E-06	0.00141212
BP	tube morphogenesis	610	194	6.12E-06	0.00147421
CC	synaptic membrane	318	115	6.79E-06	0.00161605
BP	epithelium development	815	251	7.11E-06	0.00167321
BP	positive regulation of neurogenesis	155	64	7.95E-06	0.00184904
BP	positive regulation of cell differentiation	575	183	8.14E-06	0.00186915
BP	pattern specification process	351	134	9.07E-06	0.00205891
BP	mesenchyme development	237	92	1.00E-05	0.00225278
BP	regionalization	318	123	1.03E-05	0.00227575
BP	regulation of synapse organization	214	79	1.16E-05	0.00253945
CC	postsynapse	494	158	1.28E-05	0.0027762
BP	heart morphogenesis	191	75	1.62E-05	0.00344884
BP	developmental growth	446	143	1.61E-05	0.00344884
BP	regulation of synapse structure or activity	220	80	1.80E-05	0.00377696
BP	branching morphogenesis of an epithelial tube	123	56	1.83E-05	0.00381483
BP	neural precursor cell proliferation	122	53	1.93E-05	0.00396446
BP	regulation of multicellular organismal process	1934	500	2.01E-05	0.00409751
CC	neuron to neuron synapse	270	96	2.26E-05	0.00454933
BP	regulation of epithelial cell differentiation	107	47	2.33E-05	0.00465168
BP	cell development	1835	484	2.36E-05	0.00466736
BP	roof of mouth development	70	35	2.39E-05	0.00466853
BP	positive regulation of neuron differentiation	72	38	2.41E-05	0.0046771
BP	cell fate commitment	231	96	2.79E-05	0.00536043
BP	negative regulation of neuron differentiation	53	31	2.90E-05	0.00551685
BP	enteroendocrine cell differentiation	25	18	2.98E-05	0.00560507
BP	embryo development	748	230	3.05E-05	0.0056376
BP	negative regulation of cell differentiation	467	149	3.05E-05	0.0056376
BP	morphogenesis of a branching epithelium	141	62	3.09E-05	0.00564647
BP	determination of heart left/right asymmetry	45	25	3.12E-05	0.00566181
BP	growth	604	179	3.17E-05	0.00568893
BP	regulation of trans-synaptic signaling	353	120	3.26E-05	0.00579865
BP	regulation of anatomical structure morphogenesis	568	173	3.40E-05	0.0059941
BP	inner ear development	152	61	3.54E-05	0.00618568

BP	regulation of Wnt signaling pathway	220	80	3.70E-05	0.00636513
CC	presynapse	425	136	3.69E-05	0.00636513
BP	modulation of chemical synaptic transmission	352	119	4.24E-05	0.00717338
BP	synaptic signaling	560	178	4.25E-05	0.00717338
CC	postsynaptic membrane	227	85	4.90E-05	0.00820195
CC	postsynaptic density	233	83	5.46E-05	0.00906924
CC	plasma membrane bounded cell projection	1463	372	5.57E-05	0.0091649
CC	asymmetric synapse	245	87	5.62E-05	0.00918382
BP	morphogenesis of an epithelium	341	120	5.72E-05	0.00926029
BP	morphogenesis of a branching structure	154	65	6.21E-05	0.00994768
BP	canonical Wnt signaling pathway	208	74	6.24E-05	0.00994768
BP	negative regulation of Wnt signaling pathway	116	48	7.95E-05	0.01255906
BP	trans-synaptic signaling	542	172	8.29E-05	0.01300636
BP	digestive tract development	109	49	8.37E-05	0.01301633
BP	eye development	295	102	8.71E-05	0.01344937
BP	sensory system development	304	105	9.93E-05	0.01521455
BP	telencephalon regionalization	12	11	1.04E-04	0.01579111
BP	synapse organization	386	124	1.07E-04	0.01613201
CC	distal axon	189	66	1.09E-04	0.0163763
BP	positive regulation of synapse assembly	53	26	1.15E-04	0.01708836
BP	heart looping	42	23	1.24E-04	0.01794418
CC	cell projection	1529	385	1.24E-04	0.01794418
CC	glutamatergic synapse	369	118	1.24E-04	0.01794418
BP	visual system development	297	102	1.22E-04	0.01794418
BP	embryonic heart tube morphogenesis	45	24	1.28E-04	0.01809246
BP	chemical synaptic transmission	536	169	1.27E-04	0.01809246
BP	anterograde trans-synaptic signaling	536	169	1.27E-04	0.01809246
BP	response to growth factor	483	147	1.32E-04	0.01849343
BP	mesenchymal cell differentiation	190	72	1.45E-04	0.02023478
BP	positive regulation of cell projection organization	224	74	1.47E-04	0.02025488
BP	digestive system development	118	51	1.49E-04	0.02038067
BP	type B pancreatic cell development	14	11	1.52E-04	0.02073401
BP	regulation of axonogenesis	101	41	1.56E-04	0.02114455
BP	behavior	478	150	1.58E-04	0.02120917
BP	Wnt signaling pathway	291	97	1.59E-04	0.02125155
BP	ear development	171	65	1.63E-04	0.02145001
CC	postsynaptic specialization	257	89	1.63E-04	0.02145001
BP	neuroepithelial cell differentiation	37	22	1.65E-04	0.02165247
BP	circulatory system development	798	230	1.74E-04	0.02261395
BP	metanephric nephron development	29	19	1.85E-04	0.02385466
BP	epithelial cell differentiation	490	152	1.99E-04	0.0254758
BP	neuron fate commitment	62	34	2.15E-04	0.02737398
MF	DNA-binding transcription factor activity, RNA polymerase II-specific	797	244	2.17E-04	0.02746747
BP	regulation of animal organ morphogenesis	64	32	2.25E-04	0.02829878
BP	cell fate specification	97	46	2.30E-04	0.0287021
BP	cellular response to growth factor stimulus	465	141	2.34E-04	0.02910589
BP	positive regulation of epithelial cell differentiation	48	24	2.40E-04	0.02964352
BP	positive regulation of developmental growth	114	45	2.42E-04	0.02964352
BP	regulation of synapse assembly	120	46	2.47E-04	0.03018437
BP	heart development	423	133	2.73E-04	0.03306107
BP	signal release	347	109	2.88E-04	0.03466741
CC	cell body	377	116	2.90E-04	0.03466741
BP	regulation of canonical Wnt signaling pathway	167	59	3.21E-04	0.03826216
BP	regulation of neural precursor cell proliferation	69	33	3.32E-04	0.03928092
BP	type B pancreatic cell differentiation	20	14	3.38E-04	0.03980617
BP	negative regulation of multicellular organismal process	783	215	3.53E-04	0.0412299
BP	synapse assembly	192	69	3.76E-04	0.04347169
BP	regulation of developmental growth	208	72	3.87E-04	0.04416333
BP	smooth muscle contraction	83	32	3.96E-04	0.04498321
BP	transforming growth factor beta receptor superfamily signaling pathway	237	77	4.14E-04	0.04674571
BP	cell surface receptor signaling pathway	1745	436	4.18E-04	0.04695463
CC	axon terminus	86	35	4.22E-04	0.04711586
BP	embryonic heart tube development	60	28	4.35E-04	0.04833826
BP	embryonic limb morphogenesis	96	43	4.44E-04	0.04847492
BP	embryonic appendage morphogenesis	96	43	4.44E-04	0.04847492
BP	anatomical structure formation involved in morphogenesis	810	233	4.44E-04	0.04847492
CC	neuron projection terminus	96	38	4.53E-04	0.04917666
BP	endocrine system development	103	47	4.61E-04	0.0497999

BP	export from cell	619	171	4.66E-04	0.0497999
MF	sequence-specific double-stranded DNA binding	890	260	4.64E-04	0.0497999

Supplementary Table 4. Promoter CpG cassettes identified as linked to immune infiltration by the Boruta algorithm.

Promoter cassette	Total CpGs in cassette	Overlapping genes
Cassette 9	46	*GBP4, RSAD2, RTP4, SAMD9L, AGBL3, CYREN, TMEM140, CD274, LIPA, BATF2, CARD16, OAS2, XAF1, ZBP1, APOL3, APOL1*
Cassette 24	23	*OPRM1, CBLN1, SHISA6, DNAH9*
Cassette 55	15	*PARP10, IFITM1, BST2, BISPR, MVB12A*
Cassette 4	112	*C1orf162, CTSS, ATP8B2, SLAMF7, ARHGAP25, ARHGAP15, ICOS, CXCR2, SP140L, GZMA, ZNF366, BRD8, RIPOR2, ZC3H12D, CNKSR3, SYTL3, TAGAP, AOAH, SLC26A4-AS1, GIMAP8, GIMAP6, CHRNA6, TMEM71, SRGN, CCDC88B, KRTAP5-9, CD69, PLCB2, ATP8B4, CALML4, SIRPB2, SIRPB1*
Cassette 7	51	*PDYN_PDYN-AS1 CNR2, TNFSF8, SPI1, ITGB7, SP7, PLCB2, LAT_SPNS1, SLX1A-SULT1A3_LOC388242_NPIPB12_BOLA2_SULT1A3_SLX1B, MIR4315-1_GNGT2, MIR4315-1_ABI3, MIR4315-1_CD79B, SCN1B, RIPOR3, LIME1, CBSL_U2AF1L5_CRYAA2_CRYAA_U2AF1_CBS, CBSL_U2AF1L5_CRYAA2_CRYAA_MX2_U2AF1_CBS"*
Cassette 29	20	*CD74*
Cassette 30	20	*MSX2, COL5A1, DIO3*
Cassette 3	113	*C1orf158, NUDC, CLCA1, TNFSF18, KISS1, TOGARAM2, CAPN13, COX7A2L, LRRC31, TMEM212-AS1, IDUA, GBA3, LOC105377267, ALB, DNAH5, TIFAB, GPLD1, CDHR3 CHRNA2, LRRC26, MSS51, TDRD1, SLC22A10, MIR100HG_BLID, MIR100HG, NINJ2-AS1_NINJ2, NINJ2-AS1 C12orf50, STON2, SEPTIN12_ROGDI, SMIM22_ROGDI, CRYM_CRYM-AS1, SBK2, CBSL_U2AF1L5_CRYAA2_CRYAA_U2AF1_CBS_DOP1B", CBSL_U2AF1L5_CRYAA2_CRYAA_TFF3_U2AF1_CBS, CBSL_U2AF1L5_CRYAA2_CRYAA_U2AF1_CBS, CBSL_U2AF1L5_CRYAA2_CRYAA_TFF1_U2AF1_CBS"*
Cassette 31	19	*TBR1, HOXA2*
Cassette 16	26	*MATK*
Cassette 17	25	*FAM124B, SYN2, DAPP1, ARL11*
Cassette 37	19	*ZSCAN23*

LEGENDS SUPPLEMENTARY DATA FILES

Supplementary Data File 1.

Identified CpG cassettes for all CpGs obtained from adjusted beta values using β=7 is available as *supplementary_file_1.csv*.

Supplementary Data File 2.

Identified CpG cassettes for promoter CpGs obtained from adjusted beta values using β=7 is available as *supplementary_file_2.csv*

Supplementary Data File 3.

Identified CpG cassettes for proximal CpGs obtained from adjusted beta values using β=7 is available as *supplementary_file_3.csv*.

Supplementary Data File 4.

Identified CpG cassettes for distal CpGs obtained from adjusted beta values using β=7 is available as *supplementary_file_4.csv*.

Supplementary Data File 5.

CosMx spatial transcriptomics data required for the analyses performed in this work is available as *supplementary_file_5.csv*.
